# Molecular investigation of Tuscan sweet cherries sampled over three years: gene expression analysis coupled to metabolomics and proteomics

**DOI:** 10.1038/s41438-020-00445-3

**Published:** 2021-01-01

**Authors:** Roberto Berni, Sophie Charton, Sébastien Planchon, Sylvain Legay, Marco Romi, Claudio Cantini, Giampiero Cai, Jean-Francois Hausman, Jenny Renaut, Gea Guerriero

**Affiliations:** 1 Department of Life Sciences, University of Siena, via P.A. Mattioli 4, I-53100 Siena, Italy; 2 TERRA Teaching and Research Center, Gembloux Agro-Bio Tech, University of Liège, 5030 Gembloux, Belgium; 3 Environmental Research and Innovation Department, Luxembourg Institute of Science and Technology, 41, Rue du Brill, L-4422 Belvaux, Luxembourg; 4 Environmental Research and Innovation Department, Luxembourg Institute of Science and Technology, 5, rue Bommel, L-4940 Hautcharage, Luxembourg; 5 Istituto per la BioEconomia (IBE CNR), Dipartimento di Scienze BioAgroAlimentari, via Aurelia 49, 58022 Follonica, Italy

**Keywords:** Metabolomics, Gene expression analysis, Secondary metabolism, Proteomics

## Abstract

Sweet cherry (*Prunus avium* L.) is a stone fruit widely consumed and appreciated for its organoleptic properties, as well as its nutraceutical potential. We here investigated the characteristics of six non-commercial Tuscan varieties of sweet cherry maintained at the Regional Germplasm Bank of the CNR-IBE in Follonica (Italy) and sampled ca. 60 days post-anthesis over three consecutive years (2016-2017-2018). We adopted an approach merging genotyping and targeted gene expression profiling with metabolomics. To complement the data, a study of the soluble proteomes was also performed on two varieties showing the highest content of flavonoids. Metabolomics identified the presence of flavanols and proanthocyanidins in highest abundance in the varieties Morellona and Crognola, while gene expression revealed that some differences were present in genes involved in the phenylpropanoid pathway during the 3 years and among the varieties. Finally, proteomics on Morellona and Crognola showed variations in proteins involved in stress response, primary metabolism and cell wall expansion. To the best of our knowledge, this is the first multi-pronged study focused on Tuscan sweet cherry varieties providing insights into the differential abundance of genes, proteins and metabolites.

## Introduction

*Prunus avium* L. is a fruit-tree belonging to the genus *Prunus* within the Rosaceae family that produces stone fruits with a characteristic aroma and taste. This fruit-tree is native to many regions worldwide, with a preference for temperate climates like the Mediterranean area in Europe. It has a diploid genome of 16 chromosomes (2*n* = 16) and, like other members of the Rosaceae family, sweet cherry contains toxic cyanogenic glycosides^1,2^, which are present in low concentrations in the stone (0.8%)^2^.

The fruits of *P. avium* are rich sources of health-promoting compounds^3,4^ and have a moderate content of simple sugars (and therefore a low glycemic index), as well as organic acids. They are cholesterol-free, low in calories with a high content of water. These drupes are also rich sources of vitamins (notably vitamin C) and minerals (K, P, Ca, Mg).

Polyphenols and triterpenes are among the beneficial phytochemicals composing the rich palette of bioactives in sweet cherry fruits^3,5^. Triterpenes are present in the cuticle of the fruits and, more specifically, they are found almost exclusively associated with the intracuticular waxes^6,7^.

Italy is an important producer of sweet cherries which account for an important portion of the agricultural production^8–10^; therefore, this fruit-tree plays a prominent role in the agricultural and economic landscape of Italy.

Among the different Italian regions, Tuscany is known for the high quality of its food products exported worldwide (wine, oil, cheese, meat) and for specific geographic areas within its territory that have obtained the Protected Geographical Indication (IGP) label. Such an example is Lari, where a specific variety of sweet cherry is cultivated^11^, or the reference areas of Capalbio, Batignano, Campagnatico, Castiglione delle Pescaie, where the olive varieties Frantoio, Leccino, Moraiolo and Pendolino are grown^12^.

Understanding more about the physiology and bioactive contents of non-commercial sweet cherry varieties of Italian collections can inspire exploitation programs valorizing these local fruits at the regional level. Such ancient local varieties have either disappeared or have been marginalized and reduced in number to a few trees, because of the introduction of new cherry varieties in crop systems^11^. Nevertheless, they constitute an important reservoir of interesting characters (e.g. morphological, organoleptic and genetic) which can contribute to the selection of new varieties through breeding programs^13^.

We previously showed that six non-commercial varieties of Tuscan sweet cherries maintained at the Germplasm Bank of the CNR-IBE in Follonica (Grosseto, Italy) are high producers of pentacyclic triterpenes^5^, as well as phenolics^3^. We here enrich these data by using genotyping and gene expression profiling of phenylpropanoid (hereafter abbreviated PPP) biosynthetic genes, as well as untargeted metabolomics on fruits sampled at maturity during 3 years (2016–2017–2018). We additionally investigate the soluble proteomes of two varieties, Crognola and Morellona, ranking as the highest producers of phenolic compounds. A commercial variety, Durone, commonly found in Italian fruit markets, was included in the study. The molecular data obtained by including this variety allow us to have a comparison with fruits found on the market. The goal of the study is to provide molecular information on the synthesis and content of phenolic compounds in the Tuscan sweet cherries and to compare the data with those obtained for a commercial counterpart.

The data pave the way to follow-up studies focused, for example, on earlier developmental stages, or on the post-harvest stability of the Tuscan fruits, which will provide an accurate evaluation of their further economic valorization.

## Results and discussion

### Genotyping of six non-commercial Tuscan sweet cherries

**Fig. 1:**
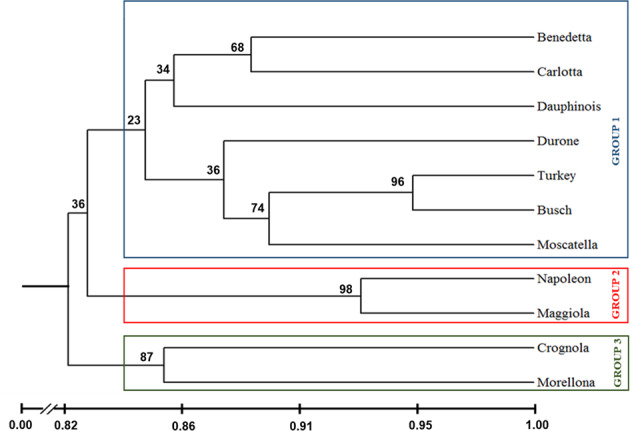
Dendrogram derived from the genotyping assay (UPGMA method) using SSR markers specific for genomic regions with a high coefficient of polymorphism. The relationships between the Tuscan cherries and the commercial ones from Luxembourg (Busch), Turkey, France (Napoleon and Dauphinois) and Italy (Durone) are shown. Nei & Li’s similarity coefficients are displayed in the black bar below the tree. Bootstrap values are indicated above the branches (1000 replicates).

As a first step towards the molecular characterization of the Tuscan sweet cherries, a similarity tree was generated (Fig. 1). Commercial varieties originating from France, Turkey and Luxembourg were included to enrich the dataset and to better discriminate the phylogenetic relatedness of the Tuscan fruits. However, these commercial varieties were not included in the other analyses performed.

In a previous study, genotyping of Tuscan sweet cherries was used to investigate the self-incompatibility alleles (*S-alleles*) which are necessary to determine incompatibility relationships between cultivars and establish appropriate breeding programs^14^. The authors focused their attention on the tree breeding incompatibility by designing SSR markers specific for the *S* locus.

Different SSR markers were here used and the results showed three main genetic clusters belonging to three different branches of the tree. The biggest cluster included three ancient varieties (Benedetta, Carlotta, Moscatella) and four commercial ones (Durone from Italy, Dauphinois from France, commercial from Turkey and commercial from Luxembourg, referred to as Busch). The second branch comprised the ancient variety Maggiola and the French one Bigarreau Napoléon (referred to as Napoleon in Fig. 1). The last cluster included the varieties Crognola and Morellona, which grouped separately from all the other Tuscan sweet cherries studied.

Despite the small number of samples here studied, genotyping was in agreement with the distribution of the varieties across the Tuscan territory: Benedetta and Carlotta share a wide distribution across the whole region, Crognola and Morellona are both from the province of Pisa, while Moscatella is the only representative of the geographical area around Siena and Maggiola of Roccalbegna (province of Grosseto).

### Untargeted metabolomics

Untargeted metabolomics identified 15 differentially abundant metabolites in positive and 14 in negative mode (Table 1 and Table 2). This approach was adopted to confirm and enrich the data already present in the literature on Tuscan sweet cherries^3^, by providing information on other families of molecules, namely flavanols, proanthocyanins and flavonolignans (cinchonain and deoxyhexosyl cinchonain; Tables 1, 2).

**Table 1:** List of differentially abundant compounds in sweet cherries obtained by UHPLC-DAD-HR-MS/MS in positive ESI mode. The details of the compounds are given, together with the specification of the reliability class and references used for the detection

Putative identification	*R*_t (min)_	Formula	Theoretical *m/z*	Observed *m/z*	Mass error (ppm)	Main MS2 fragments	MSI reliability class with references used for the annotation
Flavanol hexoside	8.79	C_21_H_24_O_11_	453.1391	453.1379	−2.78	139.0381	2^106^
A-type flavanol dimer I	13.87	C_30_H_24_O_12_	577.1341	577.1318	−3.91	245.0434	3^106^
Coumaroyl quinic acid	16.00	C_16_H_18_O_8_	339.1074	339.1065	−2.91	147.0433–119.0481–91.0534	3^107–109^
(epi)afzelechin–(epi)catechin	17.65	C_30_H_26_O_11_	563.1548	563.1534	−2.50	107.0480–147.0431–287.0544	2^110,111^
A-type flavanol trimer I	19.02	C_45_H_36_O_18_	865.1974	865.1968	−0.73	245.0441–287.0544–163.0375	3^112^
B-type flavanol trimer I	19.02	C_45_H_38_O_18_	867.2131	867.2131	0.00	245.0422–127.0379–163.0382	3^106^
B-type flavanol tetramer	19.64	C_60_H_50_O_24_	1155.2765	1155.2770	0.45	245.0440–247.0593–163.0375	3^112^
A-type flavanol trimer II	19.64	C_45_H_36_O_18_	865.1974	865.1962	−1.41	287.0539–247.0587–135.0428	3^112^
A-type flavanol tetramer	19.95	C_60_H_48_O_24_	1153.2608	1153.2609	0.06	135.0441–123.0416–163.0376	3^112^
B-type flavanol trimer II^a^	19.98	C_45_H_38_O_18_	867.2131	867.2117	−1.59	127.0374–163.0394–135.0409	3^112^
Trihydroxyflavanone	20.79	C_15_H_12_O_5_	273.0758	273.0751	−2.31	153.0167	2
B-type flavanol dimer	21.52	C_30_H_26_O_12_	579.1497	579.1492	−0.90	123.0429–127.0378–163.0331	3^112^
A-type flavanol dimer II	21.52	C_30_H_24_O_12_	577.1341	577.1320	−3.56	123.0431–245.0442–135.0430	3^108^
Cinchonain	24.52	C_24_H_20_O_9_	453.1180	453.1168	−2.67	191.0333–163.0362	3^110^
Dihydroxyflavanone hexoside	25.74	C_21_H_22_O_10_	435.1286	435.1273	−2.85	169.0122–273.0757	2^107^

All observed ions are [M+H]^+^

*R*_t_ retention time, *MSI* Metabolomics Standards Initiative

^a^Only in harvests 2016 and 2017

**Table 2:** List of differentially abundant compounds in sweet cherries obtained by UHPLC-DAD-HR-MS/MS in negative ESI mode. The details of the compounds are given, together with the specification of the reliability class and references used for the detection

Putative identification	*R*_t (min)_	Formula	Theoretical *m/z*	Observed *m/z*	Mass error (ppm)	Main MS2 fragments	MSI reliability class with references used for the annotation
A-type flavanol dimer I	13.85	C_30_H_24_O_12_	575.1195	575.1176	−3.28	125.0249–163.0017–255.0300	3^111,112^
B-type flavanol trimer I^a^	13.93	C_45_H_38_O_18_	865.1985	865.1976	−1.07	125.0231–161.0243–407.0770	3^107,112^
Coumaroyl quinic acid	15.99	C_16_H_18_O_8_	337.0929	337.0928	−0.16	173.0462–93.0341–119.0494	3^107–109^
(epi)afzelechin–(epi)catechin	17.63	C_30_H_26_O_11_	561.1402	561.1381	−3.81	289.0733–245.0821–203.0747	2^110,111^
A-type flavanol trimer I	19.01	C_45_H_36_O_18_	863.1829	863.1813	−1.79	285.0370–125.0265–161.0259	3^112^
B-type flavanol trimer II	19.01	C_45_H_38_O_18_	865.1985	865.1988	0.35	125.0244–407.0797–161.0239	3^112^
B-type flavanol tetramer^a^	19.63	C_60_H_50_O_24_	1153.2619	1153.2570	−4.27	125.0279–243.0303–161.0254	3^112^
A-type flavanol trimer II	19.63	C_45_H_36_O_18_	863.1829	863.1810	−2.16	125.0230–161.0276–243.0317	3^112^
B-type flavanol pentamer	19.93	C_75_H_62_O_30_	1441.3253	1441.3218	−2.44	125.0219–243.0327–287.0709	3^112^
Trihydroxyflavanone	20.78	C_15_H_12_O_5_	271.0612	271.0605	−2.58	153.0167	2
B-type flavanol dimer	21.50	C_30_H_26_O_12_	577.1352	577.1342	−1.61	125.0237–289.0737–161.0258	3^107,108,112^
A-type flavanol dimer II	21.50	C_30_H_24_O_12_	575.1195	575.1179	−2.79	125.0224–161.0292–177.0172	3^108^
Deoxyhexosyl cinchonain	24.53	C_30_H_30_O_13_	597.1614	597.1587	−4.43	341.0686–189.0171–217.0126	3^110^
Dihydroxyflavanone hexoside	25.72	C_21_H_22_O_10_	433.1140	433.1125	−3.40	271.0612–243.0652	2^107^

All observed ions are [M-H]

*R*_t_ retention time, *MSI* Metabolomics Standards Initiative

^a^Only in harvests 2016 and 2017

**Fig. 2:**
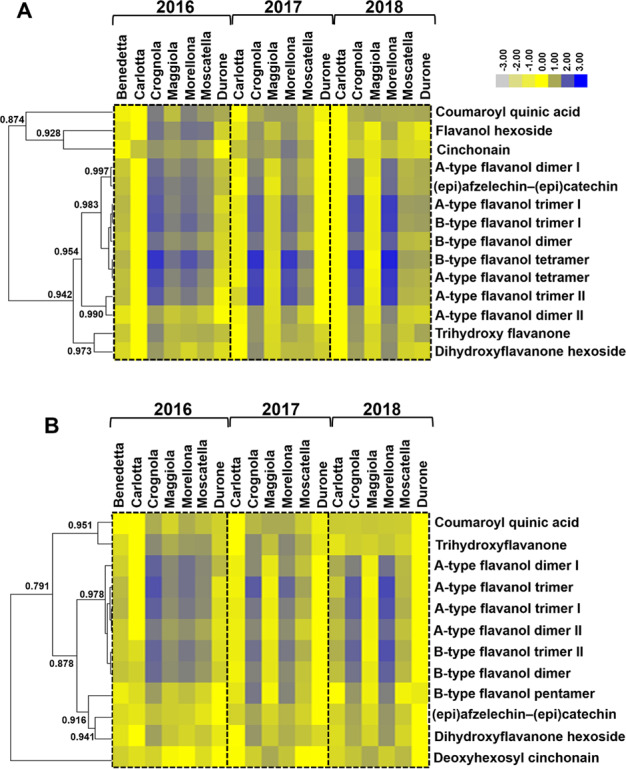
Heatmap hierarchical clustering showing the fold change differences of the compounds identified. **A** Metabolites identified in positive and (**B**) in negative mode in the 3 years. A maximum fold change >3 in absolute value was used, together with a *p*-value < 0.05 at the one-way ANOVA. Fold-changes were calculated using the means of normalized abundances. To build the heatmap, the fold change values were rescaled based on the lowest value detected per single metabolite and then log10-transformed. Numbers indicate the Pearson correlation coefficients. The color bar indicates the log10-transformed fold change values.

A hierarchical clustering of the heatmap was performed to identify similar patterns of abundance shared by the classes of molecules detected (Fig. 2). It should be noted that the variety Benedetta only appears in 2016, as the trees did not give any fruits in the other years studied.

As previously reported in other studies focused on sweet cherries^15^, the majority of the molecules detected in the Tuscan fruits belonged to the flavonoid class.

Flavonoids play different roles in plants, e.g. interaction with pollinators^16^, photoprotection, reactive oxygen species (ROS) scavenging^17^, response to abiotic stresses^18^, as well as auxin transport^19^. Their biosynthesis is known to be affected by the genotype and the environment. A study on 27 strawberry genotypes grown in the North and South of Italy revealed a higher content of flavonols in the fruits from the northern location^20^. Additionally, autochthonous cultivars of sweet cherry from South Italy showed differences in flavonoids, thereby revealing that the genotype is responsible for statistically significant differences in the content of bioactive molecules^13^.

In the Tuscan fruits examined, flavonoids varied in abundance, notably (epi)afzelechin-(epi)catechin and A/B-type flavanols. The A- and B-type flavanols observed could be fragments of bigger polymers, such as proanthocyanidins; however, the exact number of monomers is difficult to determine due to the limit of 2000 *m/z* of the MS1 scan. For these molecules, the most striking differences were observed in two varieties, i.e. Crognola and Morellona, which ranked as the highest in abundance in all the years studied (Fig. 2). This result is in agreement with the previously published data obtained using spectrophotometric assays and targeted metabolite quantification using HPLC-DAD^3,5,21^: Crognola and Morellona produced high amounts of pentacyclic triterpenes, as well as anthocyanins and flavonoids.

In contrast, the commercial cherries were among the fruits producing the lowest amounts of flavanols. Although the post-harvest storage conditions of the commercial cherries are not known and were supposedly different from those of the Tuscan fruits, they were included for comparative purposes and as representatives of the most common cherries on the Italian market.

Fewer differences among the varieties were found for the hydroxycinnamic acid coumaroyl quinic acid (that could, however, also represent a fragment of bigger molecules, such as quinic acid esters of hydroxycinnamic acids previously detected in depitted sweet cherries^22^) and for flavanones (di- and trihydroxyflavanones). These compounds were also less abundant as compared to A/B-type flavanols (Fig. 2). It is worthy to note that untargeted metabolomics detected the presence of cinchonain and deoxyhexosyl cinchonain in the fruits of sweet cherries. These secondary metabolites are flavonolignans and have high antioxidant, hepatoprotective and antimicrobial activities^23,24^. Such molecules are rare in nature and found in species such as *Cinchona*, *Trichilia*, *Acer*, *Sorbus*^25^. However, a recent study detected cinchonain in Hungarian sour cherries^26^, thereby confirming that this flavonolignan occurs in the fruits of members within the genus *Prunus*. From a nutraceutical point of view, Olszewska and colleagues showed an antiradical capacity of cinchonain (measured with the DPPH assay) up to four times higher than (+)-catechin^25^, which is known as one of the most effective antioxidants both in vitro and in vivo^27^. Moreover, in vitro experiments showed an insulinotropic effect of cinchonain and it was thus proposed that the consumption of this molecule through the diet may be helpful for managing type 2 diabetes^28^.

Crognola and Morellona produced cinchonain at higher levels (Fig. 2), a finding confirming the high nutraceutical potential of these two Tuscan varieties. The commercial variety Durone showed among the lowest amounts of the flavonolignan.

Other phenolic compounds, namely neochlorogenic acid, catechin, chlorogenic acid, epicatechin and quercetin, were identified using standards (Supplementary Tables 1, 2 and Supplementary Fig. 1) and hence classified as MSI reliability class 1 compounds. The data obtained for these compounds are in line with those obtained previously, especially for flavonoids and confirm Crognola and Morellona as the best producers of secondary metabolites^3^.

The results obtained with metabolomics showed an impact of the genotype on the biosynthesis of flavonoids: this is well known and supported by a strong body of evidence in the literature^20,29–32^. Based on these results and those previously published^3,5,21^, Crognola and Morellona appear to be genetically predisposed to produce high amounts of secondary metabolites. Interestingly, these two varieties clustered together in a separate branch of the dendrogram (Fig. 1), a finding indicating differences at the genome-level with respect to all the others.

### Targeted gene expression analysis

Since the biosynthesis of secondary metabolites is regulated at the gene level^33^, RT-qPCR was performed to quantify the relative gene expressions of the PPP-related genes. The gene expression analysis was carried out on the 3 years of harvest 2016, 2017 and 2018 and on genes intervening in the PPP. The genes investigated were phenylalanine ammonia lyase-*PAL*, cinnamate-4-hydroxylase-*C4H*, 4-coumarate-coenzyme A ligase-*4CL*, chalcone synthase-*CHS*, chalcone isomerase-*CHI*, flavanone 3-hydroxylase-*F3H*, dihydroflavonol 4-reductase-*DFR*, anthocyanidin synthase-*ANS* and a UDP-glycosyltransferase-*UGT* (responsible for the glycosylation of anthocyanin aglycones).

**Fig. 3:**
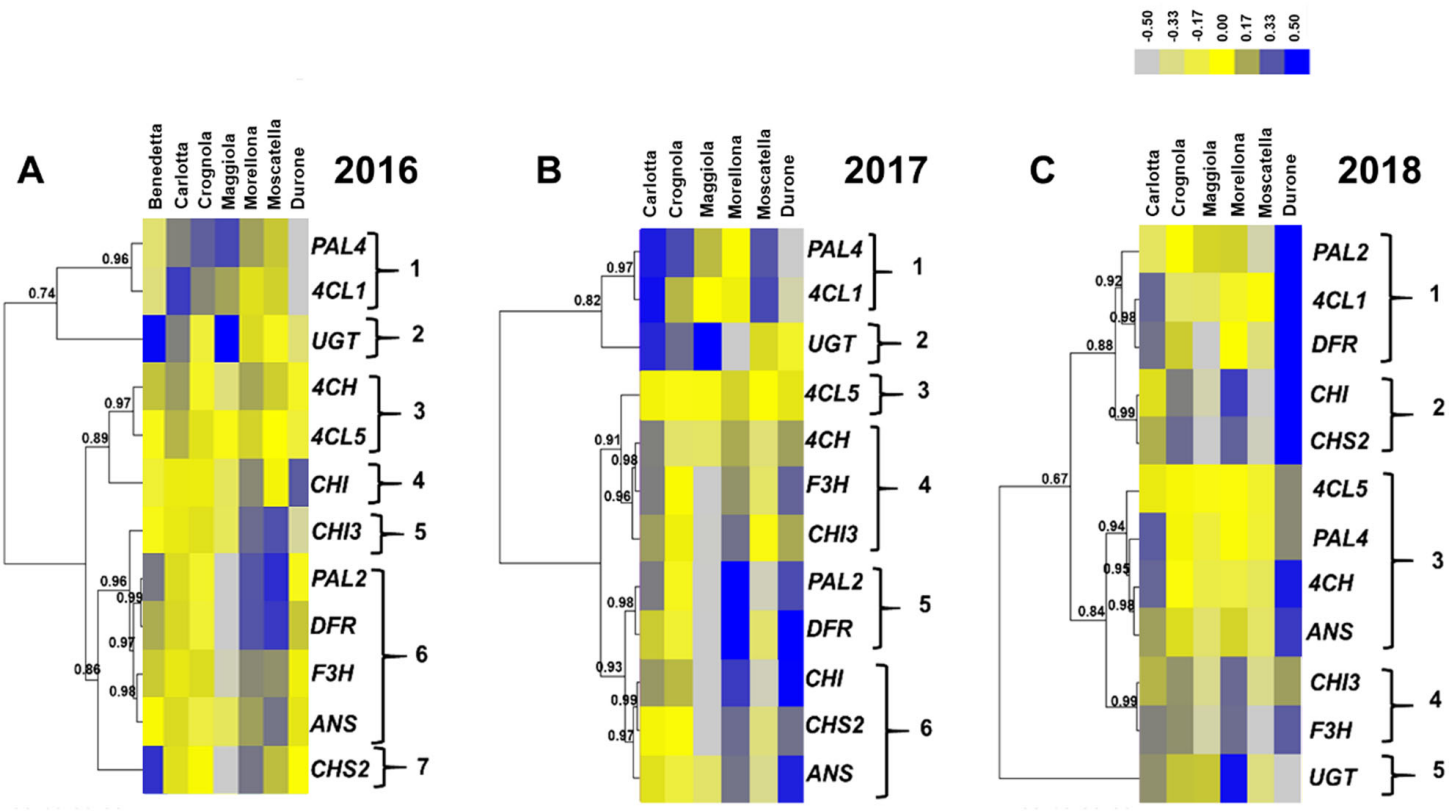
Heatmap hierarchical clustering of the PPP-related gene expression data across the 3 years of study (A, 2016; B, 2017; C, 2018). Numbers indicate the Pearson correlation coefficients. Numbers indicate the Pearson correlation coefficients. The color bar indicates the log10-transformed normalized relative quantities. The bar graphs of the expression data are available in Supplementary Fig. 2

The genes involved in the PPP are notoriously multigenic^34,35^; for *PAL*, *4CL* and *CHI*, two isoforms were analyzed because of the roles that these genes have as gatekeepers (*PAL*) and members of the general steps (*4CL*), respectively, as well as their implication in branch points (*CHI*). By studying the genes coding for isoforms, it is possible to speculate about their potential role in the provision of precursors needed for the synthesis of aromatic macromolecules. Subsequently, a hierarchical clustering of the heatmap was carried out to unveil potential correlations of expression patterns among the genes studied (Fig. 3).

In 2016, seven major patterns could be distinguished by setting 0.96 as threshold value for the Pearson correlation. The first one was composed by *PAL4* and *4CL1*, the second by *UGT*, the third by *C4H* and *4CL5*, the fourth and fifth by *CHI* and *CHI3*, the sixth comprised *PAL2*, *DFR*, *F3H* and *ANS* and the last *CHS2* (Fig. 3A). Besides *CHI*, the commercial fruits displayed lower expressions as compared to the ancient ones and this was particularly evident for the genes partaking in the general phase of the PPP, i.e. the isoforms of *PAL*, *4CL*, as well as *4CH* (Fig. 3A). A lower expression of these genes may indeed be directly responsible for a decreased synthesis of products shunted to the specialized branch of the PPP leading to the biosynthesis of flavonoids.

Carlotta, Crognola and Maggiola showed overall higher expression of *PAL4*, while *CHI3*, *PAL2*, *DFR*, *F3H*, *ANS* and *CHS2* were highly expressed in the varieties Morellona and Moscatella. The variety Benedetta showed high expression of *UGT* and *CHS2*.

In 2017, six major clusters could be recognized by setting a threshold value of 0.93 for the Pearson correlation (Fig. 3B): the first two were the same as those of 2016, i.e. *PAL4*/*4CL1* and *UGT*, the third comprised only *4CL5*, the fourth *C4H*, *F3H* and *CHI3*, the fifth cluster included *PAL2* and *DFR*, the last *CHI*, *CHS2* and *ANS*. It was possible to observe again the clustering of *PAL4* with *4CL1* and of *PAL2* with *DFR*, as previously seen for the fruits sampled in 2016. The commercial variety showed instead differences, notably higher expression of the genes involved in the central and late stages of the PPP. Morellona confirmed the high expression of genes involved in flavonoids/anthocyanin biosynthesis. Maggiola displayed a high expression of *UGT*, as previously observed in 2016 (Fig. 3A).

The year 2018 differed from the previous ones in terms of gene expression (Fig. 3C). By setting a threshold value of 0.88 for the correlation coefficient, five major expression clusters were observed: the first grouped *PAL2*, *4CL1* and *DFR*, the second *CHI* and *CHS2*, the third comprised *4CL5*, *PAL4*, *C4H* and *ANS*, the fourth cluster grouped *CHI3* and *F3H* and the last one was represented by *UGT*. *PAL2* and *DFR* were in the same cluster; however, in 2018, *4CL1* was also present, differently from the previous years, where it grouped with *PAL4*.

*PAL2* showed lower expression in Morellona with respect to 2016 and 2017, while the commercial fruits showed much higher expression of all the genes, with the exception of *UGT*.

It is interesting to note that the two *PAL* isoforms clustered with different genes in 2016 and 2017: *PAL4* grouped with *4CL1*, while *PAL2* with *DFR*. In thale cress, four different *PAL* isoforms were described^36^ and a redundant role for *PAL1* and *PAL2* was demonstrated in flavonoid biosynthesis^37^. The sweet cherry *PAL2* may be involved in the PPP branch shunting precursors towards the synthesis of flavonoids. This however awaits experimental confirmation. RT-qPCR also revealed a higher expression of the genes involved in the central and late stages of the PPP in Morellona (Fig. 3).

The expression of PPP-related genes is subjected to transcriptional regulation. V-MYB myeloblastosis viral oncogene homolog (MYB) transcription factors (TFs) are master regulators of the PPP and activate the branches leading to the biosynthesis of monolignols, flavonoids and anthocyanins^38^. Two genes encoding MYB TFs were here investigated using RT-qPCR, i.e. *MYB10.1* and *MYB11*. The choice of these genes is motivated by their role in the biosynthesis of anthocyanins^39,40^ and flavonols^41^, respectively.

**Fig. 4:**
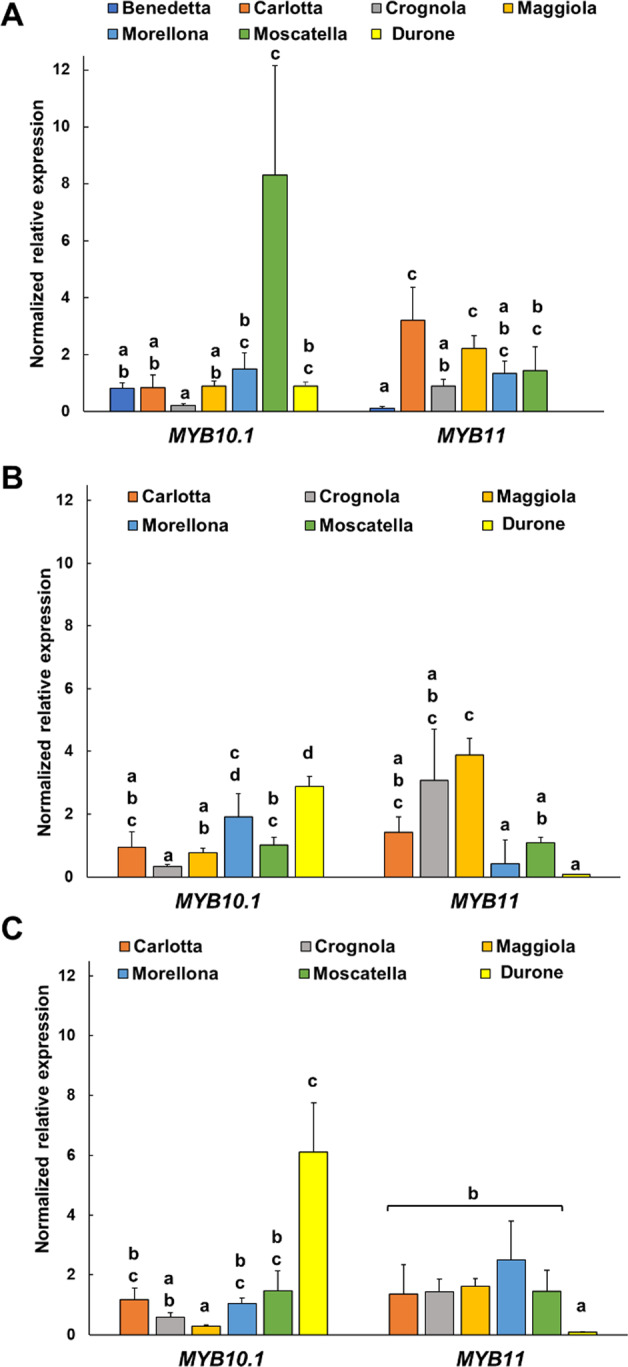
Gene expression analysis (indicated as normalized relative expression) of the MYB TF-encoding genes. **A** results relative to 2016, (**B**) results obtained on the samples harvested in 2017, (**C**) results relative to the year 2018. Error bars correspond to the standard deviation (*n* = 4). Different letters represent statistical significance (*p*-value < 0.05) among the groups of data. If a letter is shared, the difference is not significant. A one-way ANOVA followed by Tukey’s post-hoc test was performed on genes showing homogeneity and normal distribution; for the others, a Kruskal-Wallis test followed by Dunn’s post-hoc test was used. The statistical parameters in (**A**) are *MYB10.1* F(6,20) = 34.37, *p*-value = 0.000; *MYB11* F(5,18) = 28.26, *p*-value = 0.000, (**B**): *MYB10.1* F(5,18) = 35.69, *p*-value = 0.000; *MYB11 X*^2^(5) = 17.46, *p*-value=0.004 and in (**C**): *MYB10.1 X*^2^(5) = 18.44, *p*-value = 0.002; *MYB11* F(5,17) = 17.84, *p*-value = 0.000

In 2016, *MYB10.1* showed the highest expression in the varieties Moscatella and the lowest in Crognola, while in 2017 and 2018 the commercial fruits showed the highest expression of the gene (Fig. 4). Despite the variations in expression across the 3 years, Crognola was always among the varieties expressing low levels of *MYB10.1*, while Morellona showed higher expression of the transcript. Both varieties are characterized by a red color of the skin^42^; however, Morellona is the only variety with a red pulp. Therefore, the high content of anthocyanins reported previously^3,21^ can be explained by a higher gene expression. Durone showed also high expression of *MYB10.1*, especially in 2017 and 2018: this can be explained by the intense red color of both the skin and the pulp, features that make these commercial fruits particularly appealing to consumers. Although Moscatella does not display intense red pigmentation, our results show that this variety ranks among the highest producers of proanthocyanidins (Fig. 2): this result can be explained by a high expression of *MYB10.1* in this variety.

The *MYB10.1* gene was sequenced in the varieties Morellona (deposited in GenBank with the accession number MH545964) and Crognola (partial sequence in Supplementary Fig. 3) to check the occurrence of the previously reported alleles *MYB10.1a* and *b* responsible for the red and blush color of the skin^39^. The *a* allele was cloned from Crognola and the *b* allele from Morellona; it remains to be verified whether the *MYB10.1a* allele occurs together with *MYB10.1b* in Morellona, as is expected on the basis of the strong red color observed in the fruits of this variety.

The gene expression patterns of *MYB11* showed the largest variations among the Tuscan cherries in 2017, where the varieties Crognola and Maggiola were the highest and Durone the lowest. Interestingly, Durone always showed the lowest expression of the TF in all the years studied.

Overall, the RT-qPCR data showed that the PPP-related genes were differentially expressed in the Tuscan varieties. It was not possible to link the abundance of the phenolic compounds (Fig. 2, Tables 1, 2) with gene expression profiles (Fig. 3, Supplementary Fig. 2), since the highest producing varieties Crognola and Morellona did not always show high expression of PPP-biosynthetic genes. The reason for a lack of correlation may be linked to post-transcriptional and post-translational events: for example, it was reported that a Kelch repeat F-box protein, SAGL1, regulates the PPP in thale cress by interacting with PAL1 and mediating its proteasome-dependent degradation^43^. The field conditions are also known to affect the gene expression pattern and this explains the variations observed in the Tuscan fruits.

To understand the environmental causes that could be (at least in part) responsible for the differential gene expression in the Tuscan varieties, the daily temperatures from March (blooming period) to May (fruit sampling), as well as the precipitation and humidity maximum/minimum averages were retrieved from the LaMMA meteorological station in Grosseto (http://lamma.eu/en). As can be seen in Supplementary Fig. 4, the year 2018 had, in average, warmer minimum temperatures. Variations in the humidity averages were also recorded across the years, with 2016 recording lower average maximum humidity and 2018 higher average minimum humidity (Supplementary Fig. 4).

As discussed in the previous section on metabolomics, an influence of the environment on the expression of PPP-related genes is known^44^. The functional quality of strawberries was enhanced under mild drought salinity stress, since the content of phenolics, anthocyanins and ascorbic acid increased^45^. Likewise, in grapevine, seasonal water deficit was shown to affect anthocyanin biosynthesis during ripening by upregulating both genes and metabolites^46^.

The Tuscan varieties here investigated thrive in wild conditions and in soils with minimal human intervention; given the non-controlled conditions of growth, it is not surprising that gene expression showed such a high variability across the years of study.

### Analysis of the soluble proteomes of two ancient varieties

An analysis of the proteome was carried out on the varieties Morellona and Crognola. The goal was to highlight differences in the abundance of soluble proteins that could explain the metabolite and gene expression variations observed in the 3 years.

The two-dimensional difference gel electrophoresis (2D-DIGE) experiments showed 166 differentially abundant protein spots. The spots were selected according to the following parameters: max fold change >2 and *p*-value < 0.01.

The spot identification was done through peptide sequence searches in the MASCOT engine. The search was carried out against the NCBI non-redundant protein database restricted to the *P. avium* entries. From an initial number of 166 proteins, after the identification of the same protein isoforms, 51 proteins were retrieved with a good e-value and similarity sequence score, according to MASCOT (Table 3). The proteins were classified by function and category, on the basis of their known involvement in specific pathways: “Stress”, “Cell wall”, “Proteasome-related”, “Primary metabolism” and “Other” (Table 3). Considering all the proteins detected, the three categories with the highest number of differentially abundant proteins are “Stress”, “Cell wall” and “Primary metabolism” (Table 3).

**Table 3:** Details of the spot numbers, accession numbers, annotations and *p*-values of the identified proteins

Spot No	Accession No	Protein Name	Function	Category	*p*-value (year)	*p*-value (variety)
753	XP_021804554.1	Thioredoxin reductase NTRB	Oxidoreductase activity	STRESS	0.12515	**0.00297**
996	XP_021801592.1	Superoxide dismutase	Oxidoreductase activity	STRESS	**0.00965**	0.14682
1004	XP_021816866.1	2-Cys peroxiredoxin BAS1	Cell redox homeostasis/Stress response	STRESS	0.7117	**0.00026**
988	XP_021823852.1	Glutathione *S*-tr ansferase F11	Response to oxidative stress	STRESS	0.66672	**0.00001**
976	XP_021813812.1	Glutathione *S*-transferase DHAR2	Oxidoreductase activity	STRESS	**0.00007**	0.32618
1006	XP_021832122.1	Glutathione *S*-transferase	Oxidoreductase activity	STRESS	0.24001	**0.00043**
1023	XP_021808495.1	NAD(P)H dehydrogenase (quinone) FQR1	Response to auxin/Oxidoreductase activity/Stress response	STRESS	**0.00036**	0
378	XP_021806563.1	Protein disulfide isomerase	Cell redox homeostasis	STRESS	0.84562	**0**
299	XP_021815855.1	Stromal 70 kDa heat shock-related	Stress response	STRESS	0.17303	**0.00017**
948	XP_021820221.1	Low-temperature-induced cysteine proteinase	Stress response	STRESS	0.81184	**0.00003**
632	XP_021832997.1	Protein SRC2	Stress response	STRESS	0.27232	**0.00004**
845	XP_021820409.1	Glycine-rich protein 2	Response to water deficit. ABA/Cell wall	STRESS	**0.0056**	**0**
1163	XP_021824432.1	17.1 kDa class II heat shock protein	Stress response	STRESS	**0.00695**	**0.00163**
985	XP_021805019.1	20 kDa chaperonin	Stress response	STRESS	0.0128	**0.00019**
674	XP_021804100.1	Plastoglobulin-1	Lipid metabolism of chloroplasts/Stress response	STRESS	0.05678	**0.00082**
1238	XP_021831271.1	Phosphatidylglycerol/phosphatidyl inositol transfer protein	Lipid binding (recognition of pathogen related products)	STRESS	0.15061	**0.00045**
792	XP_021812280.1	Xyloglucan endotransglucosylase 31	Cell wall metabolism	CELL WALL	0.4535	**0.00001**
477	XP_021823827.1	UTP-glucose-1-phosphate uridylyltransferase	Cell wall metabolism	CELL WALL	0.40482	**0.00437**
822	XP_021802094.1	Xyloglucan endotransglucosylase 6	Cell wall metabolism	CELL WALL	0.01025	**0.00207**
347	AAA91166.1	Beta-glucosidase	Carbohydrate metabolism/Cell wall metabolism	CELL WALL	**0.00037**	0.56739
221	XP_021814417.1	Alpha-xylosidase 1	Cell wall metabolism	CELL WALL	0.08851	**0.00005**
201	XP_021823469.1	Acid beta-fructofuranosidase 1	Carbohydrate metabolism/Cell wall metabolism	CELL WALL	0.08196	**0.00561**
511	XP_021814422.1	Ubiquitin receptor RAD23c	Protein catabolic process	PROTEASOME-RELATED PATHWAY	**0.00018**	0.91013
1012	XP_021825182.1	Proteasome subunit beta type-6	Protein catabolic process	PROTEASOME-RELATED PATHWAY	0.0371	**0.00475**
936	XP_021828441.1	Proteasome subunit alpha type-6	Protein catabolic process	PROTEASOME-RELATED PATHWAY	**0.00762**	0.03074
258	XP_021832122.1	Phosphoenolpyruvate carboxykinase	Decarboxylase activity	PRIMARY METAB. GLUCONEOG.	**0.00066**	**0.00002**
161	XP_021822858.1	Aconitate hydratase	Lyase activity	PRIMARY METAB, TCA CYCLE	**0.00664**	0.41726
1453	XP_021823850.1	Isocitrate dehydrogenase	Oxidoreductase activity	PRIMARY METAB, TCA CYCLE	0.06206	**0.00213**
1293	XP_021813779.1	2-oxoglutarate dehydrogenase	Oxidoreductase activity	PRIMARY METAB, TCA CYCLE	**0.00439**	**0.00327**
709	XP_021804616.1	Malate dehydrogenase	Oxidoreductase activity	PRIMARY METAB, TCA CYCLE	**0.0004**	**0.00121**
411	XP_021825040.1	Glucose-6-phosphate 1-dehydrogenase	Oxidoreductase activity	PRIMARY METAB, PENTOSE PHOSPHATE PATHWAY	0.15166	**0.00721**
505	XP_021817392.1	6-phosphogluconate dehydrogenase. decarboxylating 3	Oxidoreductase activity	PRIMARY METAB, PENTOSE PHOSPHATE PATHWAY	0.16838	**0.00277**
883	XP_021831593.1	Gamma carbonic anhydrase 1	Carbonate dehydratase activity	PRIMARY METAB, PHOTORESP	0.39206	**0**
624	XP_021810138.1	Fructose-bisphosphate aldolase 1	Lyase activity	PRIMARY METAB, GLYCOLYSIS	0.01983	**0.00042**
251	XP_021834633.1	5-methyltetrahydropteroyltriglutamate homocysteine methyltransferase	Methyltransferase	PRIMARY METAB, AMINO ACID BIOSYNTH	0.02626	**0.000000**
209	XP_021818418.1	Eukaryotic translation initiation factor 3 subunit B	Translation regulation	PRIMARY METAB, PROTEIN BIOSYNTH	0.01917	**0.00332**
188	XP_021828563.1	Elongation factor 2	Polypeptide chain elongation	PRIMARY METAB, PROTEIN BIOSYNTH	0.06334	**0.00455**
761	XP_021821654.1	Elongation factor 1	Polypeptide chain elongation	PRIMARY METAB, PROTEIN BIOSYNTH.	0.45155	**0.00002**
910	XP_021819098.1	Soluble inorganic pyrophosphatase 4	Hydrolase activity	PRIMARY METAB, PO_4_^3-^-CONTAINING COMPOUND METABOLIC PROCESS	0.04654	**0.00001**
66	XP_021824244.1	ATP synthase subunit beta	ATP synthesis	PRIMARY METAB, ENERGY PRODUCTION	0.14432	**0.00849**
439	XP_021830782.1	Polyphenol oxidase	Oxidoreductase activity (pigment biosynthesis)	OTHER	**0.00005**	0.80611
957	XP_021826507.1	Ferritin-4	Iron binding	OTHER	**0.03802**	**0.00003**
944	XP_021820122.1	Ferritin-3	Iron binding	OTHER	**0.00021**	**0.00003**
772	XP_021825752.1	Annexin-like protein RJ4	Ca^2+^-dependent phosphlipid binding	OTHER	**0.00198**	**0**
1024	XP_021804938.1	Auxin-binding protein ABP19a	Auxin receptor	OTHER	**0.00007**	**0.00033**
887	XP_021828712.1	GTP-binding nuclear protein Ran-3	GTPase activity	OTHER	0.58551	**0.00403**
46	XP_021826137.1	Patellin-3-like	Cell cycle/cell division	OTHER	0.26719	**0.00001**
1009	XP_021804963.1	Uncharacterized protein LOC110749212	Nutrient reservoir activity /Response to ABA	OTHER	0.43022	**0.00502**
814	XP_021820843.1	Short-chain dehydrogenase TIC 32	Oxidoreductase activity	OTHER	0.14667	**0.00616**
725	XP_021806982.1	Voltage-gated potassium channel subunit beta	Ion transport	OTHER	**0.00007**	**0**
802	XP_021807453.1	Mitochondrial outer membrane protein porin of 36 kDa	Ion transport	OTHER	0.43668	**0.00712**

The *p*-values < 0.01 are in bold

**Fig. 5:**
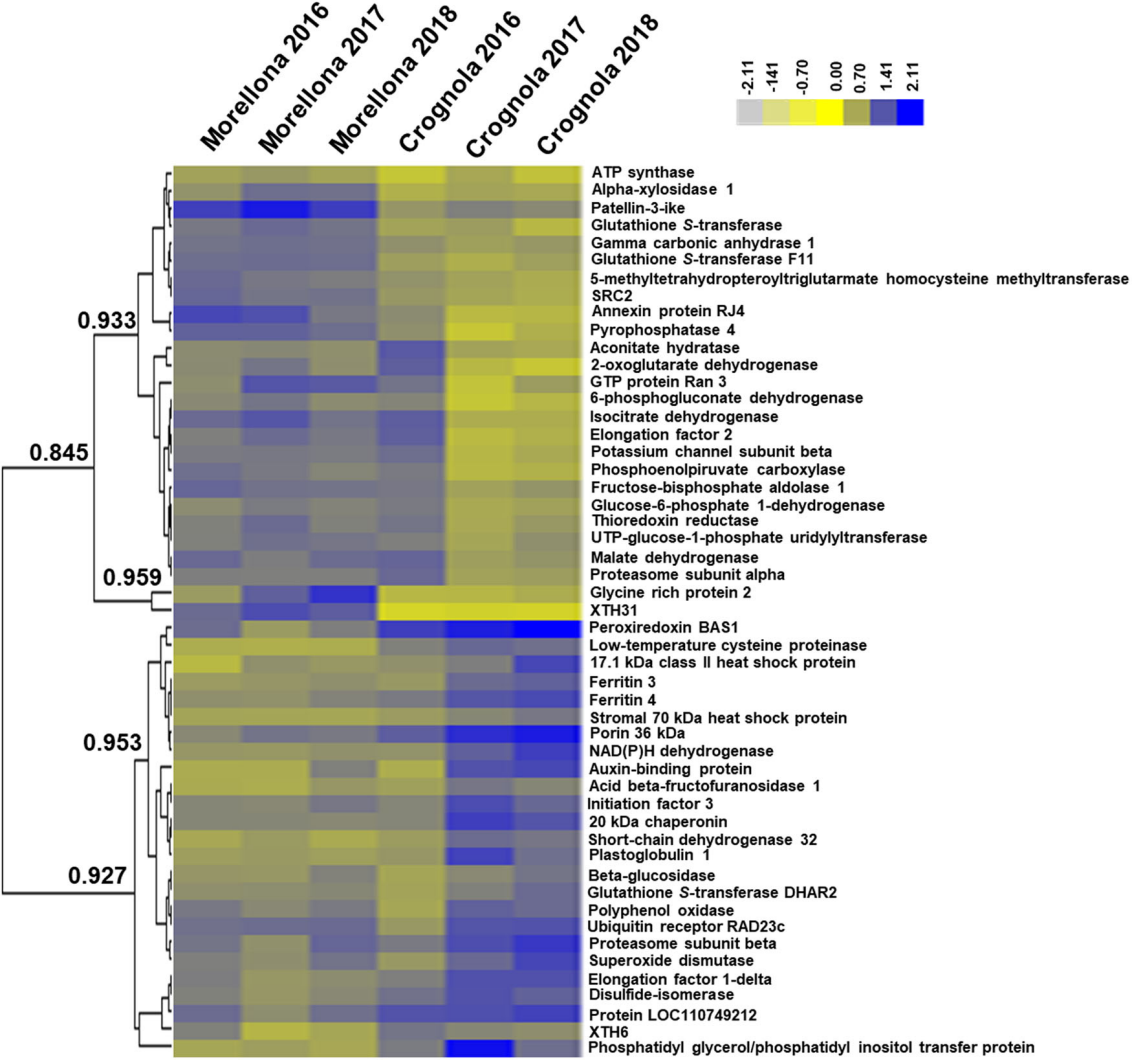
Heatmap hierarchical clustering of the 51 proteins changing significantly between Crognola and Morellona across the 3 years of study. The color bar indicates the log10-transformed relative protein abundances. The numbers indicate the Pearson’s correlation coefficients

The pattern of protein abundances between the two varieties and the 3 years is represented as a heatmap hierarchical clustering in Fig. 5. By choosing a Pearson correlation coefficient >0.94, two major clusters could be distinguished which correspond to proteins that were more abundant in Morellona or Crognola. Nevertheless, variability in the abundance of some proteins was observed in Crognola in 2016.

Hereafter, each protein category is discussed separately.

### Proteins related to stress response

In the category “Stress”, seven proteins related to the maintenance of the redox status were found, namely a thioredoxin reductase (TrxR), a superoxide dismutase (SOD), a peroxiredoxin (Prx), three glutathione-*S*-transferases (GSTs) and the quinone reductase FQR1 (Table 3). Additionally, a protein disulfide isomerase (PDI) was detected, which in plants is involved in the redox control of proteins’ disulfide bonds, thus likely acting as a chaperone in response to stress^47^. The differences were mainly related to the variety (Table 3), a finding suggesting that the two varieties respond differently to exogenous cues.

Within plant cells, during normal growth and development, there exists a balance between oxidants and antioxidants^48^. ROS are needed for normal growth but, at high levels, they cause premature senescence by oxidative stress^49^ that in fruits triggers a loss of texture, flavor and a decrease in health beneficial molecules.

Among the proteins devoted to the plant’s defense against (a) biotic stresses, glutathione *S*-transferase DHAR2 (DHAR2) is present in a similar concentration between the varieties Morellona and Crognola. On the contrary, the amount of this protein appeared to increase in relation to the different years of harvest. Indeed, in 2018 an increase of DHAR2 can be noticed in both varieties, maybe due to the variable environmental conditions. DHAR2 belongs to a subclass of enzymes in the wide group of GSTs and is able to catalyze the reduction of dehydroascorbate to ascorbate with the concomitant oxidation of reduced GSH to glutathione disulfide^50,51^.

GST F11 belongs to another GST group carrying out different reactions compared to the main GST class, due to the lack of a serine in the active site. Instead, GST F11 seems to have a role in glucosinolate metabolism^50,52^. GST F11 was highly abundant in the variety Morellona, thereby showing a dependency on the genotype. The higher abundance of GST F11, involved in the glucosinolate pathway^53^, is interesting in light of the health-related effects of isothiocyanates. These are indeed molecules obtained through the action of myrosinase on glucosinolates and displaying anticancerogenic activity^54^. It remains to be verified whether glucosinolates are present in sweet cherry (the protocol here used is indeed not optimized to extract this class of compounds) and whether Morellona produces more glucosinolates than Crognola. The differences observed may also be due to biotic stress events, since glucosinolates are typically synthesized in response to herbivores’ attack^55^.

Prx is a protein that is commonly responsible for the signaling related to ROS^56^ and acts together with TrxR and by using NADPH as a source of reducing power^57,58^. The Prx BAS1 here identified as more abundant in Crognola was reported to be involved in the protection against oxidative stress by participating, together with the Trx CDSP32, in the reduction of alkyl hydroperoxides^59^.

Statistically significant changes between the varieties were obtained also for the quinone reductase FQR1 which showed higher levels in Crognola: the corresponding gene was shown to be induced by auxin despite the absence of auxin-responsive elements in its promoter and to be involved in stress response by regulating oxidative stress together with GST^60^.

In the category “Stress”, proteins related to the response to external cues (temperature stress) were identified. More specifically, SRC2 (the product of *soybean gene regulated by cold-2*), a low-temperature-induced cysteine proteinase, a stromal 70 kDa heat shock-related protein (HSP), a 17.1 kDa class II and a 70 kDa HSPs, together with a 20 kDa chaperonin showed differences between the varieties (Table 3).

SRC2 is considered a cold stress marker: an increase of this protein was indeed reported in plant tissues exposed to low temperatures^61^.

Glycine-rich protein 2 (GRP2) was identified in 10 different spots. Two of them were more abundant in Morellona and varied across the years (Table 3, Fig. 5). Domain analysis of sweet cherry GRP2 with Motif Scan (https://myhits.isb-sib.ch/cgi-bin/motif_scan) revealed the presence of a glycine-rich, as well as, an ABA/WDS domain (Abscisic Acid/Water Deficit Stress) domain (e-value=2.9e-45). The ABA/WDS domain was found in the dual transcription factor/chaperone protein ASR1 (ABA, Stress and Ripening) which was induced in tomato upon drought and was expressed during ripening^62^. GRP2 is also linked to fruit maturation. For example, in pear, this protein increased in abundance after gibberellin application^63^. This plant growth regulator induces fruit expansion and GRPs are known to act at the cell wall level by providing a scaffold for the deposition of cell wall constituents^64^.

Crognola showed higher abundance of HSP70, 20 and 17.1 (members of small HSPs; Table 3) which are involved in the tolerance to abiotic stresses^65,66^, as well as of a low-temperature-induced cysteine proteinase showing the presence of granulin domains (e-values = 1.2e−10, 2.1e−06).

### Proteins related to the cell wall

The proteomic analysis revealed six proteins related to the cell wall: a xyloglucan endotransglucosylase/hydrolase 31 (XTH31), a UTP-glucose-1-phosphate uridylyltransferase. a xyloglucan endotransglucosylase/hydrolase 6 (XTH6), an alpha-xylosidase 1, a β-glucosidase and an acid beta-fructofuranosidase 1 (Table 3).

Xyloglucans bridge cellulose microfibril, thereby contributing to the mechanical properties of the cell walls and to morphogenesis^67^. XTHs display both xyloglucan endotransglucosylase (XET, cutting and rejoining xyloglucan chains) and xyloglucan *endo*-hydrolase (XEH, hydrolysis of xyloglucan) activities^68–71^. The majority of XTHs enzyme kinetics data showed the predominant presence of XET activity; a bioinformatic analysis coupled to structural data and enzymology predicted AtXTH31 and 32 from thale cress as potential hydrolases belonging to clade III-A^67^. Subsequent studies showed that AtXTH31 accounts for the majority of XET activity in *Arabidopsis thaliana* roots and has a pivotal role under Al stress^72^.

The phylogenetic analysis of thale cress, poplar, tomato and nasturtium XTHs showed that the cherry XTH31 clustered in group III-A, together with AtXTH31, the paralog AtXTH32 and nasturtium TmNXG1 (a predominant xyloglucan hydrolase^67^) (Supplementary Fig. 5).

It was demonstrated that thale cress XTHs from group III-A are endohydrolases involved in tissue expansion and are dispensable for normal growth^73^.

XTH31 was identified in 6 different spots; one of them showed a significant decrease in abundance in Morellona. The statistically significant difference detected between the two varieties (Table 3) is interesting if one considers the sizes of the fruits produced by the 2 varieties: Morellona is significantly bigger than Crognola (*p* < 0.05, *n* = 10, diameter Morellona = 1.77 ± 0.10 cm, diameter Crognola = 1.64 ± 0.09 cm; height Morellona = 1.95 ± 0.05 cm, height Crognola = 1.76 ± 0.10 cm).

The other XTH detected in the soluble proteomes of the two Tuscan varieties is XTH6, which clusters together with AtXTH6 (Supplementary Fig. 5). The abundance of XTH6 increased in thale cress shoots and roots under heat stress in response to cytokinin^74^, while the transcript was downregulated upon drought stress in 6 different accessions of *A. thaliana*^75^. It is therefore reasonable to assume that, in Crognola, the higher abundance of XTH6 is linked to environmental cues to which the variety reacted.

Alpha-xylosidases are involved in xyloglucan remodeling^76^; the sweet cherry XYL1 identified via proteomics is orthologous to thale cress XYL1. The higher abundance in Morellona is indicative of a higher xyloglucan remodeling at maturity. A previous study showed that *xyl1*/*axy3* mutants displayed reduced silique length and altered xyloglucan structure, where the hemicellulose was less tightly bound to other cell wall components^77^.

Differences in the abundance of a UTP-glucose-1-phosphate uridylyltransferase were also detected between the two Tuscan varieties. A BLASTp analysis against thale cress revealed sequence similarity with UGP2, one of the two genes contributing to sucrose and cell wall biosynthesis^78^. The higher expression in Morellona may indicate an involvement in cell wall-related processes and in accommodating the request of nucleotide sugars during fruit maturation. The bigger size of Morellona cherries as compared to Crognola may indeed require a higher provision of precursors for cell wall biosynthesis.

A β-glucosidase (BGLU) with sequence similarity to the apoplast-localized *A. thaliana* BGLU15 was also identified as more abundant in Crognola, with respect to Morellona.

Interestingly, despite the apoplastic localization, this protein is not related to cell wall processes, but to the hydrolysis of flavonol 3-*O*-β-glucoside-7-*O*-α-rhamnoside (a flavonol bisglycoside acting as antioxidant and reducing ROS damage), which occurs in thale cress during recovery from synergistic abiotic stresses (i.e. N deficiency, low temperature, high light intensity, UV light)^79,80^. Therefore, the BGLU protein seems to be linked to stress-related pathways.

### Proteins related to primary metabolism

In the category “Primary metabolism”, 15 differentially abundant proteins were identified (Table 3), the majority of which was more abundant in Morellona in 2017 and 2018 (Fig. 5). The differences detected may be due to the different genotypes; however, plant primary metabolism is also significantly influenced by environmental conditions, like biotic^81^ and abiotic constraints^82^ encountered in the field during the different years studied.

Three different proteins related to carbohydrate metabolism were identified (Table 3), i.e. glucose-6-phosphate dehydrogenase (G6PDH), 6-phosphogluconate dehydrogenase (6PGDH) and fructose-bisphosphate aldolase (FBA), showed significant differences between the two cherry varieties. The abundance of these 3 proteins was generally higher and steady in the variety Morellona during the 3 years. On the contrary, Crognola showed a variable abundance: indeed, as shown in Fig. 5, the levels of G6PDH, 6PGDH and FBA were higher in 2016 than 2017 and 2018. G6PDH and 6PGDH are involved in the oxidative pentose phosphate pathway and their role, despite linked to glucose oxidation, is anabolic, rather than catabolic. Additionally, G6PDH sustains nitrogen assimilation^83^ and counteracts stress conditions^84^.

Four proteins related to the tricarboxylic acid cycle (TCA) were also detected: aconitate hydratase (ACO), isocitrate dehydrogenase (IDH), 2-oxoglutarate dehydrogenase (OGDH) and malate dehydrogenase (MDH). In 2017 and 2018, the abundance of these proteins, as well as phosphoenolpyruvate carboxykinase (PEPCK), was higher in Morellona (Fig. 5). Fruit maturity is linked with primary metabolism and the production of organic acids. In sweet cherry, malate accumulates at the highest levels during stage III (coinciding with expansion and ripening) and is used for gluconeogenesis by the action of PEPCK^85^. Therefore, from the results obtained, it appears that the fruits of Morellona put in place biochemical processes linked with fruit maturation earlier than Crognola. This finding is supported by the previously described higher abundance of GRP2 which is involved in cell wall-related processes accompanying ripening, as well as of annexin RJ4 (in the category “Other”, see below), typically expressed during fruit ripening in strawberry^86^.

### Proteins related to the proteasome and other functions

In order to cope with the organism’s demands and to maintain the normal functions, cells require a continuous turnover of proteins, operated, among other actors, by the proteasome system^87^.

The ubiquitin receptor RAD23c, the proteasome subunits alpha type-6 and beta type-6 were found to be differentially abundant in dependence of the years and the varieties (Table 3).

Interestingly, for the variety Crognola, the alpha type-6 subunit was more abundant in 2016, while in 2017 and 2018, the beta type-6 subunit was higher in abundance (probably coinciding with different moments of the protein complex turnover).

It should be noted that polyphenol oxidase (PPO) was the only detected protein in the category “Other” related to the metabolism of phenolic compounds: this enzyme catalyzes the polymerization of quinones formed through the oxidation of phenols^88^ to produce brown pigments. PPO plays also a role against biotic stresses, such as insect attacks and in defense mechanisms related to altered environmental conditions^88,89^. Although a dependence on the year of harvest was detected (Fig. 5), Crognola showed the highest amount of PPO. Future studies will confirm if a higher amount of PPO in fruits may confer additional defense properties in relation to stress conditions in this variety.

Two ferritins were also more abundant in Crognola in 2017 and 2018 (Fig. 5). A ferritin was previously also identified via proteomics in peach during its development and its abundance was higher in the mesocarp^90^. Besides playing a role in iron storage, ferritins are also involved in ROS metabolism and the maintenance of the redox balance within plant tissues^91^.

Considering their secondary role in ROS detoxification, the results obtained for ferritins can be compared with those previously discussed for proteins related to the stress response. Ferritin 3 and 4 showed a higher abundance in Crognola, especially in 2017 and 2018, similarly to what described for BAS1, FQR1 and HSPs. Therefore, Crognola and Morellona showed different levels of proteins related to stress response. More specifically, Crognola had an overall higher abundance of these proteins.

### Gene expression analysis on some targets identified with proteomics

The expression of genes belonging to the categories “Stress” and “Cell wall” was measured to find a correlation with the abundances highlighted by proteomics. The expression of *PPO* within the category “Other” was also studied. Nineteen primers were designed on the targets reported in Table 4.

**Table 4:** Target proteins on whose corresponding genes primers for RT-qPCR were designed. Their categories are also indicated

Targets	Abbreviation	Category
Polyphenol oxidase	*PPO*	OTHER
SRC2	*SRC2*	STRESS
Glutathione *S*-transferase F11	*GST*	STRESS
Superoxide dismutase	*SOD*	STRESS
Thioredoxin reductase NTRB	*NTR*	STRESS
Glutathione S-transferase DHAR2	*DHAR2*	STRESS
2-Cys peroxiredoxin BAS1	*BAS1*	STRESS
Stromal 70 kDa heat shock-related protein	*70HS*	STRESS
Heat shock cognate 70 kDa protein 2	*70HS2*	STRESS
Low-temperature-induced cysteine proteinase	*LTP*	STRESS
17.1 kDa class II heat shock protein	*HSP17*	STRESS
Glycine-rich protein 2	*RBG2*	STRESS
20 kDa chaperonin	*CPN20*	STRESS
Beta-glucosidase	*BGL*	CELL WALL
Xyloglucan endotransglucosylase 31	*XTH31*	CELL WALL
Xyloglucan endotransglucosylase 6	*XTH6*	CELL WALL
Alpha-xylosidase 1	*XYL1*	CELL WALL
Acid beta-fructofuranosidase 1	*VI1*	CELL WALL
UTP-glucose-1-phosphate uridylyltransferase	*UGP2*	CELL WALL

**Fig. 6:**
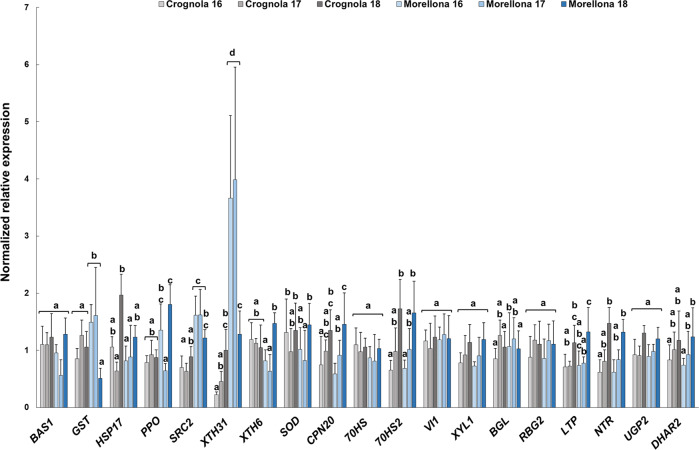
Relative expression (indicated as normalized relative expression) of some genes coding for differentially abundant proteins in the two Tuscan varieties. Error bars refer to the standard deviation (*n* = 4). Different letters represent the statistical significance (*p* < 0.05) present among the groups of data obtained. If a letter is shared, the difference is not significant. A one-way ANOVA followed by Tukey’s post-hoc test was performed on genes showing homogeneity and normal distribution; for the others, a Kruskal-Wallis test followed by Dunn’s post-hoc test was used. The statistical parameters are: *BAS1 X*^2^(5) = 2.98, *p*-value = 0.702*; GST* F(5,17) = 4.16, *p*-value = 0.012; *HSP17* F(5,17) = 7.28, *p*-value = 0.001; *PPO* F(5,17) = 9.96, *p*-value = 0.000; *SRC2* F(5,17) = 12.88, *p*-value = 0.000; *XTH31* F(5,17) = 34.95, *p*-value = 0.000; *XTH6* F(5,17) = 0.3449, *p*-value = 0.846*; SOD* F(5,17) = 6.33, *p*-value = 0.002; *CPN20* F(5,17) = 7.07, *p*-value = 0.001; *70HS X*^2^(5) = 1.067, *p*-value = 0.957; *70HS2* F(5,17) = 9.14, *p*-value = 0.000; *VI1* F(5,17) = 1.55, *p*-value = 0.225; *XYL1* F(5,17) = 1.300, *p*-value = 0.310*; BGL X*^2^(5) = 14.27, *p*-value = 0.014; *RBG2 X*^2^(5) = 4.083, *p*-value = 0.537; *LTP* F(5,17) = 8.73, *p*-value = 0.000; *NTR* F(5,17) = 4.51, *p*-value = 0.008; *UGP2* F(5,17) = 2.49, *p*-value = 0.072; *DHAR2* F(5,17) = 1.539, *p*-value = 0.230

The gene expression graph is given in Fig. 6. Hereafter, the genes confirming the trend observed in proteomics are described with more emphasis.

The gene encoding the 2-Cys peroxiredoxin BAS1 showed no difference among the three years in Crognola, while in Morellona sampled in 2017 it displayed the lowest expression. Generally, *BAS1* showed lower expression in Morellona, thereby confirming the results obtained with proteomics (Fig. 5 and Table 3).

*GST F11* showed higher expression in Morellona: despite the low expression in 2018, it was highly expressed in this variety in the years 2016 and 2017, thus following the trend of the protein.

The gene *HSP17.1* varied in expression among the years of harvest, mostly in the variety Crognola. According to Table 3, the protein HSP17.1 showed statistically significant variations across the years of study, confirming the gene expression results. Moreover, the high protein abundance in Crognola in 2018 was in agreement with the high expression in Fig. 5.

The gene *PPO* showed variations in the 3 years in Morellona, especially in 2016 and 2018. In accordance with these data, the relative protein showed statistically significant variations across the years (Fig. 5).

A clear trend could be observed for *SRC2*, expressed at higher levels in the variety Morellona. Notably, the relative protein was also more abundant in Morellona (Fig. 5).

In the category “Cell wall”, the gene coding for XTH31 was expressed at higher levels in Morellona (Fig. 6), as previously seen also for the protein abundances (Fig. 5). Differently from what observed with proteomics, *XTH6* did not show a statistically significant difference between the two varieties. In 2018, *XTH6* was induced in Morellona (Fig. 6).

The partial agreement of the RT-qPCR data with proteomics is not surprising: it is known that gene expression changes are not always accompanied by a similar trend in the corresponding proteins^92^. This can be due to post-transcriptional modifications or to other protein processing events. However, it was possible to confirm, at the gene level, that *XTH31* was upregulated in Morellona at the sampling time-point.

## Conclusions

This is the first study providing a multi-angle molecular analysis of non-commercial sweet cherry fruits from Tuscany. From the results obtained with metabolomics, it emerges that the Tuscan sweet cherries are interesting from a nutraceutical point of view. In particular, the varieties Crognola and Morellona are the most valuable in terms of bioactive content and show genetic features that distinguish them from all the others. Besides being rich sources of flavonoids, the two varieties were found to produce higher amounts of the rare flavonolignan cinchonain which has interesting health properties. The RT-qPCR analysis revealed what was already shown for other fruits, i.e. that the expression of the genes differed among the varieties and across the years and could not always explain the differences observed in the content of secondary metabolites. Proteomics revealed differences between the two most interesting varieties in terms of flavonoids’ abundance, Morellona and Crognola.

Despite the differences detected across the years for gene expression, it is possible to resume the key finding as follows: (a) Morellona and Crognola showed the highest contents of phenolic compounds, (b) the higher production of metabolites in Morellona was accompanied by a high expression of genes involved in the late phases of the PPP in 2016 and 2017 and (c) the 2 top producers of phenolics show different proteome signatures, i.e. stress-related proteins were more abundant in Crognola, while Morellona showed higher abundance of proteins related with primary metabolic functions, fruit maturation and cell wall remodeling.

These data open the way to future investigations aimed at studying the Tuscan fruits at different developmental stages and/or at assessing the post-harvest stability of the cherries produced by Morellona and Crognola. It may be that the two varieties show a distinct post-harvest behavior which makes one of them more suitable for a commercial valorization.

## Materials and methods

### Sample collection

The sampling of the six Tuscan sweet cherry varieties Morellona, Moscatella, Maggiola, Crognola, Carlotta and Benedetta was carried out in the morning (between 9:00 and 10:00 am) on May the 16^th^ 2016, May 19^th^ 2017 and May 18^th^ 2018 (temperatures: min 12 °C-max 22 °C in 2016, min 13 °C-max 26 °C in 2017, min 8 °C-max 24 °C in 2018). Samples were analyzed in four biological replicates, each consisting of a pool of 4-6 fruits for a total of 20-24 fruits coming from at least 3 different trees per variety. Fruits were sampled ca. 60 dpa (days post anthesis) from the same trees for each of the years investigated. The experimental field coordinates, growth conditions and total number of trees for each variety have been previously reported^42^. All the phenotypical aspects of the genotypes are reported in the Tuscan germplasm website (http://germoplasma.regione.toscana.it/index.php?option=com_content&view=article&id=5&Itemid=110).

One variety, Benedetta, gave fruits only in 2016. The commercial variety Durone, here included for comparative purposes, was purchased at a local grocery shop in Siena. After removing the stem, the fruits were immersed in liquid nitrogen and stored at −80 °C.

### Genotyping

DNA was extracted from fruits including the exocarp and the mesocarp. The samples were reduced to a fine powder with liquid nitrogen and DNA was extracted with the QIAGEN DNeasy Plant Mini Kit, following the manufacturer’s instructions. Each sample extraction was repeated three times. After extraction, the concentration values were measured at the Nanodrop. Fourteen primer pairs were used (Table 5) and were taken from the literature^93–98^. The primers were previously tested on species belonging to the same family, i.e. *P. cerasus* (sour cherry), *P. armeniaca* (apricot) and *P. persica* (peach). SSR markers were selected for their high polymorphism (Table 5). PCR reactions were prepared in a final volume of 20 µL using genomic DNA at 2 ng/µL, Q5^®^ Hot Start High-Fidelity 2X Master Mix and 5 µM of forward and reverse primers.

**Table 5:** Sequences of the 14 primer pairs used for genotyping and relative details. Fluorophore, size range, melting temperature, number of alleles and references are detailed

Primer name	5′ labeling	Sequence (5′→3′)	Size range (bp)	*T*_m_ (°C)	Allele No	Reference
UDP98411 Fwd	Dragonfly Orange	AAGCCATCCACTCAGCACTC	155–179	57	4	^93,94^
UDP98411 Rev		CCAAAAACCAAAACCAAAGG				
UDP98412 Fwd	FAM	AGGGAAAGTTTCTGCTGCAC	110–124	57	4	^93^
UDP98412 Rev		GCTGAAGACGACGATGATGA				
UDAp420 Fwd	FAM	TTCCTTGCTTCCCTTCATTG	166–172	56	3	^95^
UDAp420 Rev		CCCAGAACTTGATTCTGACC				
BPPCT039 Fwd	Dragonfly Orange	ATTACGTACCCTAAAGCTTCTGC	135–150	55	4	^96^
BPPCT039 Rev		GATGTCATGAAGATTGGAGAGG				
AMPA101 Fwd	FAM	CAGTTTGATTTGTGTGCCTCTC	184–192	56	4	^97^
AMPA101 Rev		GATCCACCCTTTGCATAAAATC				
UDAp-414 Fwd	HEX	CAAGCACAAGCGAACAAAAT	142–146	56	3	^95^
UDAp-414 Rev		GGTGGTTTCTTATCCGATG				
UDAp-415 Fwd	Dragonfly Orange	AACTGATGAGAAGGGGCTTG	157–161	56	2	^95^
UDAp-415 Rev		ACTCCCGACATTTGTGCTTC				
UDP96008 Fwd	Dragonfly Orange	TTGTACACACCCTCAGCCTG	148–152	60	3	^93,94^
UDP96008 Rev		TGCTGAGGTTCAGGTGAGTG				
BPPCT034 Fwd	Dragonfly Orange	CTACCTGAAATAAGCAGAGCCAT	221–255	57	7	^96^
BPPCT034 Rev		CAATGGAGAATGGGGTGC				
BPPCT040 Fwd	Dragonfly Orange	ATGAGGACGTGTCTGAATGG	133–148	57	5	^96^
BPPCT040 Rev		AGCCAAACCCCTCTTATACG				
EMPaJ15 Fwd	FAM	TTTTGGTCAATCTGCTGCTG	216–253	60	6	^98^
EMPaJ15 Rev		CTCTCATCTTCCCCCTCCTC				
EMPaS11 Fwd	HEX	ACCACMGAGGAACTTGGG	59–103	60	5	^98^
EMPaS11 Rev		CTGCCTGGAAGAGCAATAAC				
EMPaS12 Fwd	FAM	TGTGCTAATGCCAAAAATACC	135–145	60	4	^98^
EMPaS12 Rev		ACATGCATTTCAACCCACTC				
UCD-CH17 Fwd	HEX	TGGACTTCACTCATTTCAGAGA	188–212	60	6	^98^
UCD-CH17 Rev		ACTGCAGAGAATTTCCACAACCA				

The PCR parameters were as follows: initial cycle at 98 °C for 30 s, 35 cycles at 98 °C for 10 s, 1 min at 60 °C and 30 s at 72 °C, final extension at 72 °C for 2 min.

PCR products were first visualized on agarose gels. Then, they were multiplexed, diluted in double distilled water (1:50 v/v) and futher analyzed on an ABI3500 Genetic Analyzer (Life Technologies, Waltham, MA, United States). Subsequently, fragment analysis was carried out using the Genemapper 5.0 software. The sweet cherry allelic profiles obtained by genotyping were used to generate a phylogenetic tree using an Unweighted Pair-Group Method with Arithmetic mean (UPGMA) and Sequential Agglomerative Hierarchal Nested (SAHN) cluster analysis with the NTSYSpc software version 2.2 (Exeter Software, USA).

### Sample preparation for untargeted metabolomics

Whole fruits without the stones were ground using a mortar and a pestle in liquid nitrogen and approximately 500 mg of frozen powders were aliquoted and lyophilized in a freeze-dryer (Christ, Osterode, Germany). Lyophilized samples were then accurately weighed (approximately 15 mg) and stored at −80 °C until extraction. Before adding the extraction solvent, 2 µL of chloramphenicol (5 mg/mL, Sigma-Aldrich), used as internal standard, were put directly on the powders and then 998 µL of the extraction solvent (MeOH:H_2_O 80%; v/v) were added to the samples. The samples were vortexed thoroughly and then shaken in a Thermomixer (Eppendorf, Hamburg, Germany) at 1400 rpm for 4 h at 21 °C. The samples were vortexed again and centrifuged for 30 min at 20000 g at 4 °C. The supernatants (750 µL) were collected and completely evaporated using a CentriVap Vacuum Concentrator (LABCONCO, Kansas City, MO, US). Finally, the samples were resuspended in 188 µL of MeOH:H_2_O 5% (v/v) with 0.1% of formic acid (FA) and filtered through 0.22 µm polytetrafluoroethylene (PTFE) filters (Merck Millipore. Darmstadt. Germany). The harvest years 2016 and 2017 were analyzed in 2018 and the 2018 harvest year was analyzed in 2019, with the same protocol and analytical conditions.

### Untargeted metabolomics analysis with UHPLC-DAD-HR-MS/MS

The separation of molecules was achieved using an Acquity UPLC I-class UHPLC (Waters, Milford, MA, US) with a PDA detector, coupled to a hybrid quadrupole-time of flight (Q-TOF) mass spectrometer TripleTOF 6600 (SCIEX Instruments, Concord, ON, Canada) with a DuoSpray Ion Source operating in negative and positive ion mode. Five µL of the samples in random order were injected in the UHPLC system and analyzed in a run time of 60 min. The separation was performed on a reverse-phase Acquity UPLC BEH C18 column (2.1 × 100 mm, 1.7-μm particle size) (Waters, Milford, MA, US). The solvents used were A) water + 0.1% FA and B) acetonitrile (ACN) + 0.1% FA, all LC-MS grade and the column was maintained at 50 °C during all the run time. The gradient was as follows: 0 min, 1% B; 4 min, 1% B; 16 min, 5% B; 35 min, 40% B; 45 min, 100% B; 50 min, 100% B; 54 min, 1% B; 60 min, 1% B, at 0.5 mL/min flow rate. UV-visible spectra were also acquired between 190 and 800 nm at a rate of 10 points/s.

The ESI parameters were set as follows: source temperature of 650 °C, ion spray voltage of −4500 V and 4500 V, for the negative and positive mode, respectively, curtain gas (nitrogen) of 30 psi, nebulizer gas (air) of 55 psi and turbine gas (air) of 50 psi. The declustering potential was set at −60 eV in negative mode and 60 eV in positive mode. The precursor charge state selection was set at 1. For information-dependent acquisition in high sensitivity mode, survey scans were acquired in 175 ms and the 10 most abundant product ion scans were collected during 200 ms if exceeding a threshold of 100 counts/s, the total cycle time being 2.225 s. A sweeping collision energy setting of 15 eV below and above 15 eV was applied to all precursor ions. The dynamic exclusion was set for 2 s after three occurrences before the precursor could be fragmented again. For MS1, full HR-MS spectra between 100 and 2000 mass-to-charge ratio (*m/z*) were recorded. MS2 scans were recorded between 50 and 2000 *m/z*, in profile mode.

### Data analysis

The software Progenesis QI (v2.3.6275.47962, Nonlinear Dynamics, Waters, Newcastle, UK) was used to generate a list of potentially differentially abundant compounds for each ionization mode (a compound in Progenesis QI is a combination of retention time and *m/z* ratio deduced from isotopes and adducts ions) for the data corresponding to 2016 and 2017. All possible adducts and automatic processing were selected, with default automatic sensitivity and no chromatographic peak width indicated.

For the positive ionization mode, the number of compounds for the 3 years of harvest was 14776, 2583 of which had experimental MS2 data. Out of these 2583 compounds, 206 respected the statistical criteria fixed. Progenesis QI gave an identification proposal for 197 compounds, but 15 metabolites were in the end identified.

For the negative ionization mode, 19295 compounds were obtained with Progenesis QI, 4350 of which had experimental MS2 data. Out of these 4350 compounds, 446 respected the statistical criteria fixed. Progenesis gave an identification proposal for 396 compounds, but 14 metabolites were in the end identified. There was no signal-to-noise selection, except for the parameter “default” for the sensitivity of peak picking in Progenesis QI.

Alignment and peak picking were done with default parameters, then all adducts between 0 and 50 min and only compounds with available MS2 data were kept for further statistical analysis. Once the statistics had been performed with R, we also took advantage of Progenesis QI plugins to tentatively identify compounds in the databases Pubchem, MassBank, NIST, ChemSpider and ChEBI, as well as in an in-house database.

R^99^ (v3.6.0 64-bit) was used to normalize the abundances using internal standard and dry weight and to perform a one-way analysis of variance (ANOVA) with genotype as a factor on the abundances of the compounds in the first two harvests data to establish a list of compounds to search in databases. The criteria of compounds’ choice were *p*-value ANOVA < 0.05 and maximum fold-change >3.

PeakView (v 1.2.0.3, SCIEX, Concord. ON, Canada) and database Metlin in addition to the other databases available in Progenesis QI were used to perform manual identification checking.

Annotations and identifications were classified in accordance with the levels of Metabolomics Standards Initiative (MSI)^100^. Compounds in class 1 were identified by comparison with standards analyzed in the same analytical conditions, based on exact mass, retention time, MS2 fragmentation pattern and UV–visible spectrum, compounds in class 2 were identified based on the same criteria by comparison with data in databases and/or literature. Class 3 was assigned to compounds with the same information as class 2 when they allowed only chemical class determination, typically when the molecule identified is a fragment of a bigger not fully determined molecule.

Calculation of fold-changes and average abundance per genotype were performed again on the three harvests for the already putatively identified molecules when harvest 2018 data were available.

The hierarchical clustering of the heatmaps relative to metabolomics, as well as gene expression and proteomics were obtained with Cluster 3.0 (available at: http://bonsai.hgc.jp/~mdehoon/software/cluster/software.htm) and Java TreeView (available at: http://jtreeview.sourceforge.net/).

Raw data were deposited in the repository MetaboLights, under the study number MTBLS1803 (http://www.ebi.ac.uk/metabolights/).

### Bioinformatics and primer design

The genes of interest were obtained by blasting the thale cress protein sequences in NCBI, as well as by querying the Genome Database for Rosaceae (GDR; available at https://www.rosaceae.org/) and the cherry database (available at http://cherry.kazusa.or.jp). Multiple alignments were performed in CLUSTAL-Ω (http://www.ebi.ac.uk/Tools/msa/clustalo/). The primers were designed with Primer3Plus (http://www.bioinformatics.nl/cgi-bin/primer3plus/primer3plus.cgi) and checked with OligoAnalyzer 3.1 (http://eu.idtdna.com/calc/analyzer). All the primers and their relative features were previously described^5^, or are reported in Table 6. The maximum likelihood phylogenetic tree of XTHs was constructed from full-length protein sequences from sweet cherry and other species, namely poplar, tomato, thale cress, nasturtium^67^. The sequences were aligned with CLUSTAL-Ω and the tree obtained by using IQ-TREE web server (http://iqtree.cibiv.univie.ac.at/) with the “Auto” parameter to identify the best-fit substitution model^101^. The tree was rooted with *Bacillus licheniformis* lichenase (accession no CAA40547).

**Table 6:** List of primers used for gene expression analysis. Details relative to the sequences of the target genes, together with primers’ amplification efficiency %, melting temperature, amplicon sizes and accession numbers are provided

Name	Primer sequence (5′ →3′)	*T*_m_	Efficiency (%)	Amplicon size (bp)	Accession number
		(°C)			
*Phenylpropanoid pathway*					
PavPAL2 Fwd	CTGCGAGGGAAAGATTATCG	83.9	92.04	114	XM_021948624
PavPAL2 Rev	AGTGGAATGGAATGCAGCAC				
PvPAL4 Fwd	AGCCTCTTCCTTTCCCATTC	79.1	95.88	155	XM_021971014
PvPAL4 Rev	AATGCCAAACTTGACGAACC				
Pav4CH Fwd	TCCGCATTTTTCCTCTGC	84.8	88.32	111	GU990522.1
Pav4CH Rev	ATGATGGCGATGAAGAGACC				
Pav4CL2 Fwd	GTTGCGATGCCGTATTCTTC	85.7	100.45	105	XM_021954366.1
Pav4CL2 Red	TTCTCCCCATCCACTTGTTG				
Pav4CL5 Fwd	TGATGGTGAGGAAGGAAAGG	84.2	109.59	147	XM_021954558.1
Pav4CL5 Rev	TCAGATTCTTGTGCGACGAC				
PavCHS2 Fwd	GTACCAACAAGGCTGTTTTGC	88.1	93.28	146	KP347499.1
PavCHS2 Rev	TGTCAAGGTGGGTATCACTGG				
PavCHI3 Fwd	ATAGATTGGCAGCCGATGAC	80.9	91.46	143	XM_021945901.1
PavCHI3 Rev	AATCTCAGCAGTGGCAGAAG				
PavCHI Fwd	TTTCCACCGTCAGTCAAACC	85.7	96.42	102	KP347511.1
PavCHI Rev	TCACGAAGTTCCCCTGAATC				
PavF3H Fwd	GAAGATTGTGGAGGCTTGTG	78.8	87.1	72	XM_021960102.1
PavF3H Rev	ATGAGCTTGGCATCAACTCC				
PavDFR Fwd	GCCCATTTCTCATGTCATCC	81.9	86.6	116	XM_021975874.1
PavDFR Rev	TCGTCCAAGTGAACGAACTG				
PavANS Fwd	ATGGGCAGTTTTCTGTGAGC	83.27	88.78	100	XM_021947877.1
PavANS Rev	GTTCTTGGTGGGAAGATTGG				
PavUGT Fwd	ACAACTTGGGCACCTCAAAC	81.26	93.55	80	XM_021947368.1
PavUGT Rev	AGTGAACTCCAACCGCAATG				
PavPPO Fwd	ATGCGAGCCTTACCAGATG	86.07	110.51	104	XM_021975090.1
PavPPO Rev	AGATCCGAATACCCGACTTG				
*Cell wall*					
PavBGL Fwd	AGAATGGCATGGACGAGTTC	79.64	92.17	97	XM_021950416.1
PavBGL Rev	TAACAGAGGTGGCGATAGCAG				
PavXTH31 Fwd	TCTCTGGTTTGACCCAACAC	78.84	98.12	95	XM_021956588.1
PavXTH31 Rev	TTCTTACGGGCACATCATCC				
PavXTH6 Fwd	GTGCGTGATGAGCTAGACTTTG	81.92	96.09	103	XM_021946402.1
PavXTH6 Rev	TTTGCTCCCTGTTACCCTTC				
PavXYL1 Fwd	CTTCCTCAACCCGAAAACTG	82.32	91.02	100	XM_021958725.1
PavXYL1 Rev	AGCCTCGTTCATGTCAATCC				
*Stress response*					
PavVI1 Fwd	CCTGCTTGGTTGGATCAATG	80.71	91.66	83	XM_021967777.1
PavVI1 Rev	TCCTTGGAATGGTCTGAAGG				
Pav70HS Fwd	TGATGAGGTGGAAAGGATGG	82.05	92.87	93	XM_021960163.1
Pav70HS Rev	TCAGCCTGGTTCTTTGTGTC				
Pav70HS2 Fwd	TGGGAGGAGAGGATTTTGAC	78.97	95.27	91	XM_021958066.1
Pav70HS2 Rev	CTTGGGTTTCCGATGATGTC				
PavUGP2 Fwd	TCGTCTCTCGTTATGTCAGTGG	81.38	96.85	104	XM_021968135.1
PavUGP2 Rev	AGGTGCCAAGCCATCATAAG				
PavSRC2 Fwd	TGACGTTAAGGTTACGATTAGGG	85.67	98.85	108	XM_021977305.1
PavSRC2 Rev	GGCGGAGCAGGATAATCTC				
PavLTP Fwd	TGTATTTACGGGACGGTGTG	80.45	94.21	108	XM_021964529.1
PavLTP Rev	CCACCCCATGAATTTCTCAC				
PavGST Fwd	TATTGGGAACAACCCTGGAG	81.65	96.09	108	XM_021968160.1
PavGST Rev	GCACCAGAAGTTGAAGTACCAG				
PavHSP17 Fwd	AGGACGACAATGTGCTTCTG	83.66	96.02	107	XM_021968740.1
PavHSP17 Rev	TAAACTTGCCGACTCTCCTCTC				
PavRBG2 Fwd	CGAGTCAAAGAAAGACCCAGAG	84.2	92.14	104	XM_021964717.1
PavRBG2 Rev	GATGCTCATGGAAGGCAAAC				
PavSOD Fwd	AGAAGCACCAGACTTACG	84.47	82.12	92	XM_021945900.1
PavSOD Rev	AACAACAGCAGCAGCATCAC				
PavCPN20 Fwd	TCAAGGTTGCTGAGGTTGAG	82.32	90.81	82	XM_021949327.1
PavCPN20 Rev	GTGCCAATCGAAGGTTTCTC				
PavNTR Fwd	GAGCAACCCGAAAATCAGAG	84.2	85.51	107	XM_021948862.1
PavNTR Rev	CCCCAGTCACCAAATTCTTC				
PavDHAR2 Fwd	CTCAGCGACAAACCCAAATG	82.59	104.4	97	XM_021958120.1
PavDHAR2 Rev	TCACGTCAGAATCAGCCAAC				
PavBAS1 Fwd	GTTTGCCCCACAGAAATCAC	82.32	90.81	106	XM_021961174.1
PavBAS1 Rev	CAAGGTGCGAAAACACACTG				
*MYB transcription factors*					
PavMYB10.1 Fwd	TTAGGTGACGAGGATGCTTT	81.12	107	132	KP455680.1
PavMYB10.1 Rev	TTAGTCCTTCTGAACATTGG				
PavMYB11 Fwd	TTGTCGAAGCAGGACATGAG	83.93	108	73	XM_021949436.1
PavMYB11 Rev	TCCTCCCACAACCAAGAAAG				

### RNA extraction, reverse transcription and RT-qPCR

RNA extraction from whole fruits comprising the exocarp and the mesocarp (excluding the stones), purity/integrity measurement, cDNA synthesis and RT-qPCR were performed as previously described^42^. A melt curve analysis was performed at the end of the PCR cycles to check the specificity of the primers. The primers’ amplification efficiencies were determined using a calibration curve consisting of a serial dilution of 6 points (10–2–0.4–0.08–0.016–0.0032 ng/μL).

The expression values were calculated with qBase^PLUS^ (version 3.2, Biogazelle, Ghent, Belgium)^102^ by using *PavACT7* and *PaveTIF4E* as reference genes, which were sufficient for normalization according to geNORM^103^.

The log10-transformed NRQ (Normalized Relative Quantities) results were analyzed with IBM SPSS Statistics v20 (IBM SPSS, Chicago, IL, USA). Normal distribution of the data was checked with a Shapiro-Wilk test and graphically with a Q-Q plot. Homogeneity was checked with the Homogeneity of Variance Test. For data following normal distribution and homogeneous, a one-way ANOVA with Tukey’s post-hoc text was performed. For data not following normal distribution and/or not homogeneous, a Kruskal-Wallis test was performed with Dunn’s post-hoc test.

### Cloning and sequencing of *MYB10.1*

Genomic DNA was extracted from the fruits (devoid of the stones) of Crognola and Morellona using the QIAGEN DNeasy Plant Mini Kit, as previously described.

PCRs were performed using 50 ng DNA and the Q5 Hot Start High-Fidelity 2X Master Mix, following the manufacturer’s instructions. The final volume of the reactions was 50 µL and the primers were used at the final concentration of 0.5 µM. The PCR program consisted of an initial denaturation at 98 °C for 1 min, followed by 35 cycles at 98 °C for 10 s, 62 °C for 30 s and 72 °C for 1 min, then a final extension at 72 °C for 2 min was performed and the reaction kept at 4 °C. The following primers were used: MYB10.1 Fwd ATGGAGGGCTATAACTTGGGTG MYB10.1 Rev TTAGTCCTTCTGAACATTGGTACA. PCR products were run on a 2% agarose gel, then they were purified using the PCR purification kit from QIAGEN, following the manufacturer’s instructions. The eluted products were ligated into the pGEM-T Easy vector, according to the manufacturer’s recommendations and cloned into JM109 chemically competent cells. Three positive clones for each variety were grown o/n at 37 °C in LB medium supplemented with ampicillin 100 μg/ml. The following day, plasmids were extracted with the QIAGEN plasmid miniprep kit and sequenced on an Applied Biosystems 3500 Genetic Analyzer using the BigDye Terminator v3.1 Cycle Sequencing and the BigDye XTerminator Purification kits, according to the manufacturer’s instructions.

### Protein extraction

Whole cherry fruits comprising the exocarp and the mesocarp (excluding the stones) were ground in liquid nitrogen with a mortar and a pestle. The fine powder (1 g) was resuspended in 1 mL of cold acetone with 10% of trichloroacetic acid (TCA) and 0.07% of dithiothreitol (DTT)^104^. The samples were vortexed and left at -20 °C for 60 min, then centrifuged 5 min at 10000 g. The pellets thus obtained were washed twice with cold acetone and, then, dried at room temperature overnight. The dried pellets were resuspended in 0.8 mL of phenol (Tris-buffer, pH 8.0) and 0.8 mL of SDS buffer [30% (w/v) sucrose, 2% (v/v) SDS, 0.1 M Tris-HCl pH 8.0, 5% (v/v) 2-mercaptoethanol]. The mixtures were thoroughly vortexed and centrifuged for 3 min at 10000 g. The upper phase (300 µL) was transferred in a new 2 mL-tube, diluted in 5 volumes of cold ammonium acetate (NH_4_CH_3_CO_2_) in MeOH and left at -20 °C for 30 min. The precipitated samples were washed twice with the same solution, removing the supernatants each time. Finally, the samples were washed with 80% (v/v) acetone 2 times and the pellets were dried. The dried pellets were dissolved in a buffer of urea 7 M, thiourea 2 M, Tris 30 mM and CHAPS 4% (w/v). The extracted proteins were quantified using the Bradford method^105^, using BSA for the standard curve.

### 2D-DIGE

A volume of sample equivalent to 50 µg of proteins was labeled for DIGE analysis. The biological replicates of each sample were split and marked half with CyDye 3 fluorochrome and half with CyDye 5. The CyDye 2 fluorochrome was added to the internal standard, which is a mixture of all samples in equal amount. The labeling was done by the addition of 400 pmol of dye, followed by a 30 min incubation on ice in the dark. Then, 1 µL of lysine 10 mM was added to stop the reaction and the samples were incubated 10 more min in the same conditions. The samples were combined as follows: 1 Cy3-labeled, 1 Cy5 labeled and 1 internal standard. They were then loaded on strips (pH 3-10 nonlinear, 24 cm) for the first dimension, using the passive rehydration method. Nine µL of ampholytes and 2.7 µL of destreak reagent were added to 450 µL of the sample in buffer solution [urea 7 M, thiourea 2 M, Tris 30 mM and CHAPS 0.5% (w/v)]. The strips were rehydrated with the samples overnight. The isoelectric focusing (IEF) was performed with an Ettan IPGphor 3 system (GE Healthcare). A gradual increase of the voltage was used to reach a total of ca. 90000 V h within 25 h, through 5 steps planned as follows: 0–3 h 100 V, 3–7 h ramping to 1000 V, 7–14 h 1000 V, 14–20 h ramping to 10000 V and 20–25 h 10000 V. The second dimension was run on precast 12% flatbed gels (25×20 cm) in a horizontal electrophoresis tower (HPE FlatTOP Tower, Serva) following the manufacturer’s instructions. The 2D gels were scanned using a laser scanner (Typhoon FLA 9500, GE Healthcare) and consequently analyzed with the software SameSpots (http://totallab.com/home/samespots/).

### Spot picking and mass spectrometry

Spots of interest were selected using the SameSpots program using two filters, *p-*value < 0.01 and max fold change >2 (a total of 166 proteins was obtained). The gel spots were picked with an Ettan Spot Picker (GE Healthcare) and trypsinized using an EVO2 workstation (Tecan). The dried samples were solubilized in 0.7 µL of an α-cyano-4-hydroxycinnamate solution (7 mg/mL in 50% acetonitrile and 0.1% trifluoroacetic acid) and spotted onto a MALDI plate. A MALDI mass spectrum was acquired using the SCIEX 5800 TOF/TOF (Sciex). The 10 most intense peaks, excluding known contaminants, were automatically selected and fragmented. MS and MS2 were submitted to an in-house MASCOT server (version 2.6.1; Matrix Science, www.matrixscience.com) for database-dependent identifications against the NCBI non-redundant protein sequence database (NCBInr) limited to the taxonomy *P. avium* (taxID4229; 10 July 2019; 35758 sequences). The parameters were as follows: peptide mass tolerance 100 ppm, fragment mass tolerance 0.5 Da, cysteine carbamidomethylation as fixed modification (alkylation was performed during the equilibration step between IEF and second dimension) and methionine or tryptophan oxidation, double oxidation of tryptophan and tryptophan to kynurenine as variable modifications. Kynurenine, resulting from tryptophan oxidation, is an artifact often observed during automatic digestion in the laboratory where the analysis was performed (Luxembourg Institute of Science and Technology-LIST). Up to two miscleavages were allowed. All identifications were manually validated.

The mass spectrometry proteomics data have been deposited to the ProteomeXchange Consortium via the PRIDE partner repository with the dataset identifier PXD019468.

## Supplementary information

Suppl. material
