# Supplementary material for: Molecular investigation of Tuscan sweet cherries sampled over three years: gene expression analysis coupled to metabolomics and proteomics
Source: Hortic Res. 2021 Jan 1;8:12. doi: 10.1038/s41438-020-00445-3 (PMC7775447; doi:10.1038/s41438-020-00445-3)
Supplement: Supplementary file 1 — Suppl. material [file 41438_2020_445_MOESM1_ESM.pdf]

Supplementary material

Supplementary Table 1. List of differentially abundant class 1 compounds in sweet cherries obtained by UHPLC-DAD-HR-MS/MS in positive ESI mode. The details of the compounds are given; R_t, retention time. MSI, Metabolomics Standards Initiative. All observed ions are $[M+H]^+$. Catechin, quercetin and neochlorogenic acid were purchased from Sigma-Aldrich (St Louis, MO), epicatechin from Phytoplan Diehm & Neuberger GmbH (Heidelberg, Germany) and cholorogenic acid from Acros Organics (Geel, Belgium).

**Compounds confirmed by standard**	$R_{t\ (min)}$	**Formula**	**Theoretical *m/z***	**Observed *m/z***	**Mass error (ppm)**	**Main MS2 fragments**	**MSI reliability class**
**Neochlorogenic acid**	5.07	$C_{16}H_{18}O_9$	355.1024	355.1013	-3.03	163.0382	1
**Catechin**	8.00	$C_{15}H_{14}O_6$	291.0863	291.0858	-1.61	139.0378 – 123.0427	1
**Chlorogenic acid**	10.06	$C_{16}H_{18}O_9$	355.1024	355.1011	-3.41	163.0384	1
**Epicatechin**	14.95	$C_{15}H_{14}O_6$	291.0863	291.0862	-0.38	139.0380 – 123.0428	1
**Quercetin**	24.91	$C_{15}H_{10}O_7$	303.0499	303.0493	-1.96	303.0521	1

Supplementary Table 2. List of differentially abundant class 1 compounds in sweet cherries obtained by UHPLC-DAD-HR-MS/MS in negative ESI mode. The details of the compounds are given; R_t, retention time. MSI, Metabolomics Standards Initiative. All observed ions are $[M-H]^-$. Catechin, quercetin and neochlorogenic acid were purchased from Sigma-Aldrich (St Louis, MO), epicatechin from Phytoplan Diehm & Neuberger GmbH (Heidelberg, Germany) and cholorogenic acid from Acros Organics (Geel, Belgium).

**Compounds confirmed by standard**	$R_{t\ (min)}$	**Formula**	**Theoretical *m/z***	**Observed *m/z***	**Mass error (ppm)**	**Main MS2 fragments**	**MSI reliability class**
**Neochlorogenic acid**	5.07	$C_{16}H_{18}O_9$	353.0878	353.0878	0.00	191.0568	1
**Catechin**	7.93	$C_{15}H_{14}O_6$	289.0718	289.0716	-0.84	203.0722 – 245.0822 – 109.0303	1
**Chlorogenic acid**	10.00	$C_{16}H_{18}O_9$	353.0878	353.0876	-0.57	191.0539	1
**Epicatechin**	14.92	$C_{15}H_{14}O_6$	289.0718	289.0722	1.41	245.0828 – 203.0720 – 289.0736	1
**Quercetin**	24.88	$C_{15}H_{10}O_7$	301.0354	301.0343	-3.66	150.9981	1

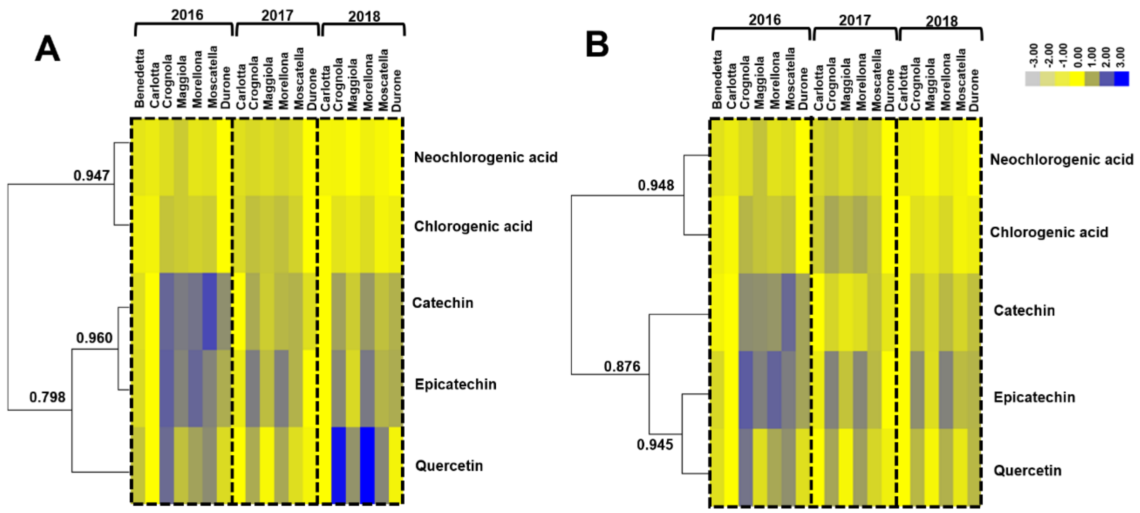

Supplementary Figure 1. Heat map hierarchical clustering showing the fold change differences of the identified compounds (class 1 compounds). (A) Metabolites identified in positive and (B) in negative mode in the three years. To build the heatmap, the fold change values were rescaled based on the lowest value detected per single metabolite and then log10-transformed. Fold-changes were calculated using the means of normalized abundances. Numbers indicate the Pearson correlation coefficients. The color bar indicates the log10-transformed fold change values.

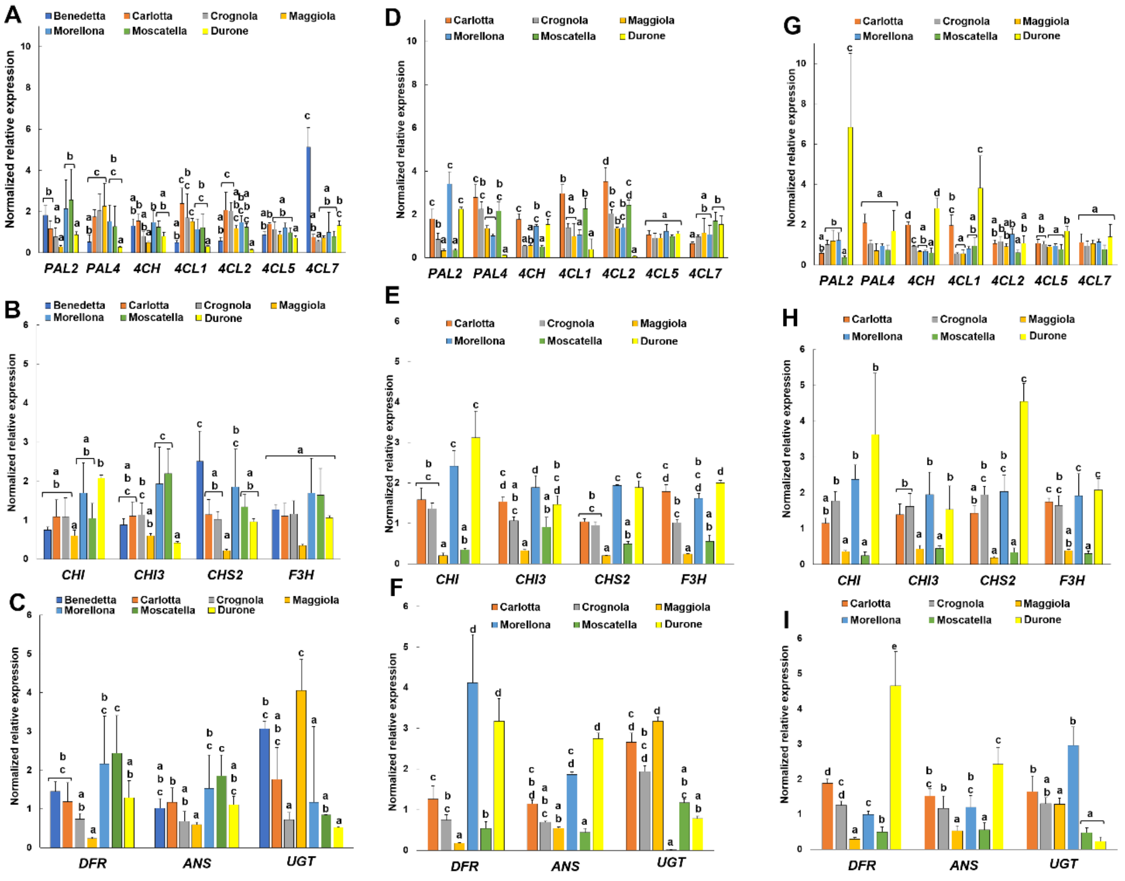

Supplementary Figure 2. Expression data (indicated as Normalized relative expression) of the genes intervening in the upper, intermediate and late steps of PPP in 2016 (A-C), 2017 (D-F) and 2018 (G-I). Error bars refer to the standard deviation (n=4). Different letters on the vertical bars indicate statistically significant differences (p<0.05) among groups. If a letter is shared, the difference is not significant. A one-way ANOVA followed by Tukey's post-hoc test was performed on genes showing homogeneity and normal distribution; for the others, a Kruskal-Wallis test followed by Dunn's post-hoc test was used. The statistical parameters in A are *PAL2* $X^2(6)=14.22$, p-value=0.027; *PAL4* F(6,19)=4.73, p-value=0.004; *4CH* F(6,20)=5.38, p-value=0.002; *4CL1* $X^2(5)=16.31$, p-value=0.006; *4CL2* $X^2(6)=19.50$, p-value=0.003; *4CL5* F(6,20)=3.30, p-value=0.019; *4CL7* $X^2(6)=15.90$, p-value=0.014; in B *CHI* F(6,20)=3.23, p-value=0.022; *CHI3* F(6,20)=6.57, p-value=0.001; *CHS2* $X^2(6)=17.47$, p-value=0.008; *F3H* $X^2(6)=11.35$, p-value=0.078; in C *DFR* $X^2(6)=15.05$, p-value=0.020; *ANS*

$X^2(6)=14.52$, *p*-value=0.024; *UGT* $X^2(6)=17.48$, *p*-value=0.008; in D *PAL2* $F(5,18)=34.54$, *p*-value=0.000; *PAL4* $X^2(5)=20.47$, *p*-value=0.001; *4CH* $X^2(5)=18.28$, *p*-value=0.003; *4CL1* $X^2(5)=18.80$, *p*-value=0.002; *4CL2* $X^2(5)=21.20$, *p*-value=0.001; *4CL5* $F(5,18)=0.854$, *p*-value=0.533; *4CL7* $X^2(5)==12.85$, *p*-value=0.025; in E *CHI* $X^2(5)=21.13$, *p*-value=0.001; *CHI3* $X^2(5)=20.53$, *p*-value=0.001; *CHS2* $X^2(5)=21.25$, *p*-value=0.001; *F3H* $X^2(5)=21.31$, *p*-value=0.001; in F *DFR* $F(5,18)=72.39$. *p*-value=0.000; *ANS* $X^2(5)=21.12$, *p*-value=0.000; *UGT* $X^2(5)=22.40$, *p*-value=0.000; in G *PAL2* $F(5,17)=19.08$, *p*-value=0.000; *PAL4* $X^2(5)=14.22$, *p*-value=0.079; *4CH* $F(5,17)=31.69$, *p*-value=0.000; *4CL1* $X^2(5)=16.31$, *p*-value=0.006; *4CL2* $F(5,17)=5.25$, *p*-value=0.004; *4CL5* $F(5,17)=5.33$, *p*-value=0.004; *4CL7* $X^2(5)=14.22$, *p*-value=0.079; in H *CHI* $X^2(5)=20.09$, *p*-value=0.001; *CHI3* $F(5,17)=17.48$, *p*-value=0.000 *CHS* $X^2(5)=19.93$, *p*-value=0.001; *F3H* $X^2(5)=16.07$, *p*-value=0.007; in I *DFR* $F(5,17)=80.67$, *p*-value=0.000; *ANS* $F(5,17)=12.41$, *p*-value=0.000; *UGT* $X^2(5)=19.15$, *p*-value=0.002.

```
ATGGAGGGCTATAACTTGGGTGTGAGAAAAGGAGCTTGGACTAAAGAGGAAGATGATGTTTTGAGGCAGTGCATT
GAGAATCATGGAGAAGGAAAGTGGTACCAAGTTCCTTACAAAGCAGGTATTAATGTAAATATAGCTCAAAGAGAG
ATATATGATATAGATTGTTCATATATAGCTAGAGCTTAATAGGCAGTGAAGCCTTAATTAAATTAGTGATGTGTA
CAAGGTCTTAAACTTCTCCACTTATTGCCAATTGGTTGCTTTTAATTTTGTCTTTCACCAGCTAGGCCCAATAGT
CACTAGTGCTCCTATATTATTCACTATTTATGTTGTCTCCGTGTTTAGATCAAATGATATTAGTCGTGGATCGAG
TGGTGCTAATAGACTTATCCCACATCGGGAATTTTTTGCATTATGCATGGATGCAGGGTTGAACAGGTGCAGGAG
GAGCTGTAGACTAAGGTGGTTGAACTATTTGAAGCCAAATATCAAGATAGGAGGGTTTGCAGAGGATGAAGTAGA
CCTAATAATTAGGCTTCACAAGCTTTTAGGAAACAGGTACCAATAAATACGTGTCTCTTTCCTTATCCCACATCG
TTCTTTCATCACATACCATTCAAAAAAAATAAAAATAAAATACACAATCGCCGACAAGCATCCCGTGCTTTGTTT
TCTATATTATATCTTCTGTTGTTTATCTCAGTACGCATGCACAACCACAAAAAGCACTAGAAGGGCCATGTAGCC
ATGCATGATGCATCTTAGTCTCTGTGAATCGTAAAACATAGTGATCATATATATATATGTTAGAACATTTACAGA
AAGGTTTAAATTTGTGTCTGAAAGAACATGGGGTAAACTCAACAATGCTTCTTATGGAAGATAAACATCTACTGT
GGTTATTCAATTACAACAACTTGGATCTGCAAATTCCTTCTACTATATAAGCTCTAACCTGTGGTTTGATGAAGA
AAATAAACTTATAAAAGCGTAGTATCTGAAAGTTTACGTGAATCTTCCAACAAAACTAACAATCCCTTTCATAGC
AGCTGAAACATAAACCTCATTTGTTGGAAAAACATCACATCTTTCTAACTTTTTTTTGGTGTATCATAGCACAAA
TGTATTGATTTATTTTCTCACGCTATCTGTCGAAGGTGGTCATTGATTGCTGGAAGGCTTCCAGGAAGGACAGCG
AATGATGTGAAAAATTATTGGAACACTCGACTGCGGACGGATTCTCGCCTGAAAAAGGTGAAAGATAAACCCCAA
GAAACAATAAAGACCATCGTAATAAGACCTCAACCCCAAAGCTTCATCAAGAGTTCAAATTGTTTGAGCAGTAAA
GAACCAATTTTGGACCATATTCAAACGGTCGAGAATTGTAGTACGCCATCACAAACATCACCATCAACAAAGAAT
GGA
```

Supplementary Figure 3. Partial sequence of *MYB10.1a* cloned from Crognola. In yellow highlight introns. In cyan highlight, insertion not present in *MYB10.1b* from Morellona (accession number MH545964).

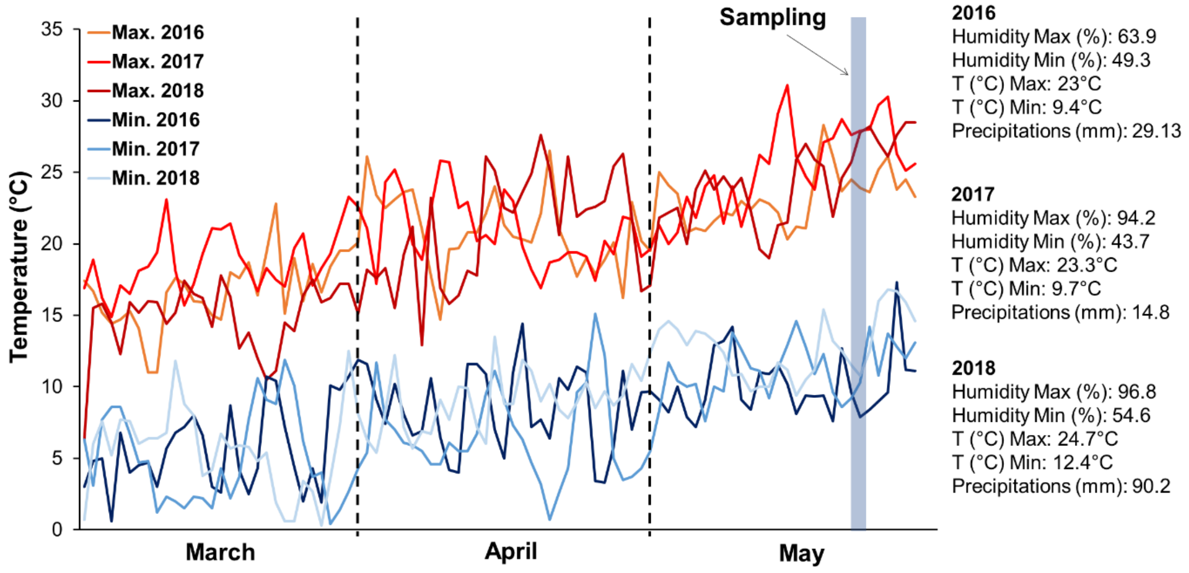

Supplementary Figure 4. Meteorological parameters registered by LaMMA station. Temperature profiles (maximum and minimum) reported by the LaMMA station placed in the experimental field and relative to March-May for the tree years. The averages of maximum and minimum temperatures are shown in the graphs, as well as the average of max/min humidity (%) and precipitations (mm). The grey bar shows the harvest time-window.

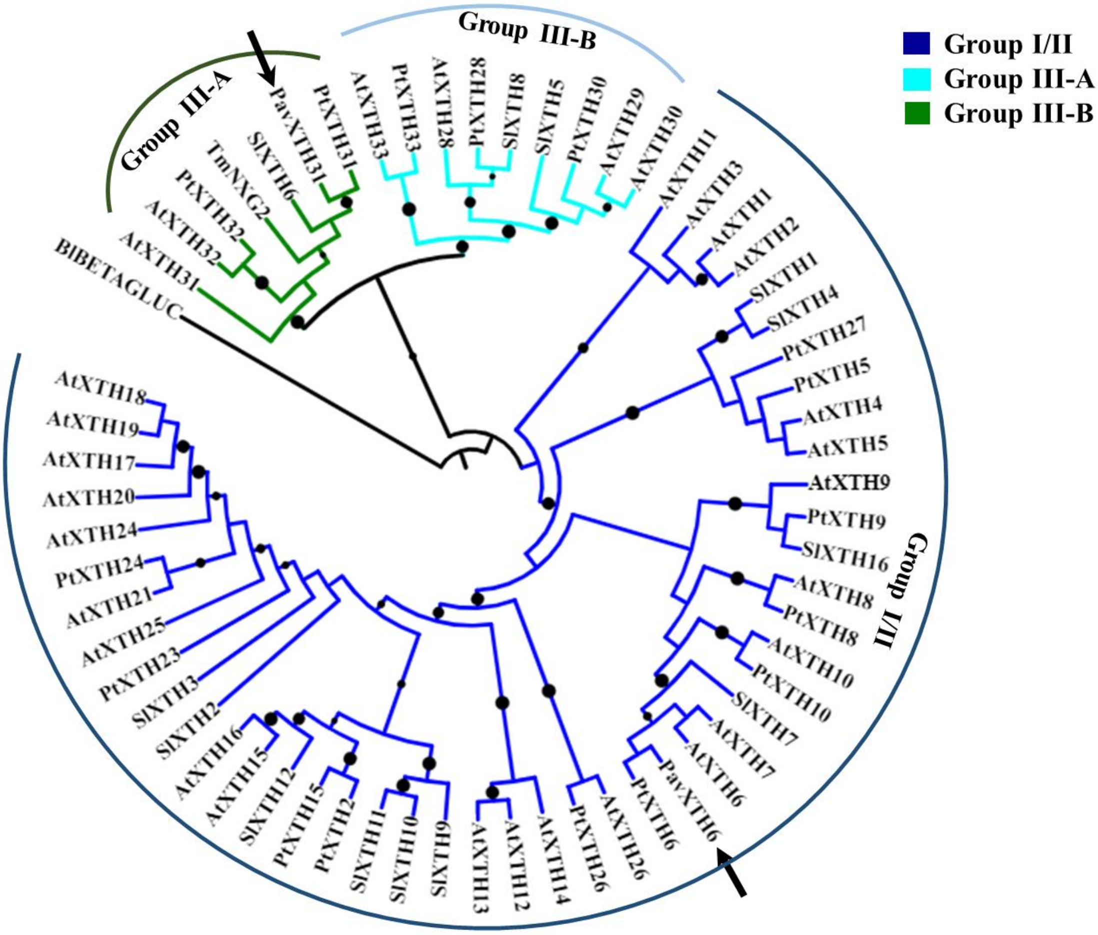

Supplementary Figure 5. Maximum-likelihood phylogenetic tree of poplar, nasturtium, thale cress and sweet cherry XTHs. The tree is rooted with a bacterial lichenase (CAA40547). Bootstraps=1000. Circles indicate bootstrap values $\geq$80%. The bigger the circles, the higher the bootstrap values. The arrows indicate the sweet cherry XTHs detected with proteomics.
